# Metal–Organic‐Framework‐Derived Nanostructures as Multifaceted Electrodes in Metal–Sulfur Batteries

**DOI:** 10.1002/adma.202008784

**Published:** 2021-05-25

**Authors:** Rui Yan, Tian Ma, Menghao Cheng, Xuefeng Tao, Zhao Yang, Fen Ran, Shuang Li, Bo Yin, Chong Cheng, Wei Yang

**Affiliations:** ^1^ College of Polymer Science and Engineering State Key Laboratory of Polymer Materials Engineering Department of Ultrasound West China Hospital Sichuan University Chengdu 610065 China; ^2^ State Key Laboratory of Advanced Processing and Recycling of Non‐ferrous Metals Lanzhou University of Technology Lanzhou Gansu 730050 P. R. China; ^3^ Functional Materials Department of Chemistry Technische Universität Berlin Hardenbergstraße 40 10623 Berlin Germany; ^4^ Department of Chemistry and Biochemistry Freie Universität Berlin Takustrasse 3 14195 Berlin Germany

**Keywords:** energy storage materials, metal‐organic frameworks, metal–sulfur batteries, nanostructures, porous carbon, polysulfide catalysts, polysulfide electrodes

## Abstract

Metal‐sulfur batteries (MSBs) are considered up‐and‐coming future‐generation energy storage systems because of their prominent theoretical energy density. However, the practical applications of MSBs are still hampered by several critical challenges, i.e., the shuttle effects, sluggish redox kinetics, and low conductivity of sulfur species. Recently, benefiting from the high surface area, regulated networks, molecular/atomic‐level reactive sites, the metal‐organic frameworks (MOFs)‐derived nanostructures have emerged as efficient and durable multifaceted electrodes in MSBs. Herein, a timely review is presented on recent advancements in designing MOF‐derived electrodes, including fabricating strategies, composition management, topography control, and electrochemical performance assessment. Particularly, the inherent charge transfer, intrinsic polysulfide immobilization, and catalytic conversion on designing and engineering of MOF nanostructures for efficient MSBs are systematically discussed. In the end, the essence of how MOFs’ nanostructures influence their electrochemical properties in MSBs and conclude the future tendencies regarding the construction of MOF‐derived electrodes in MSBs is exposed. It is believed that this progress review will provide significant experimental/theoretical guidance in designing and understanding the MOF‐derived nanostructures as multifaceted electrodes, thus offering promising orientations for the future development of fast‐kinetic and robust MSBs in broad energy fields.

## Graphical abstract

Recent advancements in designing metal–organic framework (MOF)‐derived nanostructures for multifaceted electrodes in metal–sulfur batteries (MSBs) are presented. Particularly, the inherent charge transfer, polysulfides immobilization and conversion, and morphology–property correlations for MSB applications are systematically discussed. The future tendencies regarding the construction of MOF‐derived nanostructures are also summarized, which will provide experimental/theoretical guidance in developing fast‐kinetic and robust MSBs.

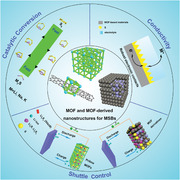

## 1 Introduction

The accelerated increase of the global energy requirements has created the urgency to develop reproducible energy sources and sustainable storage technologies to tackle the two critical issues of the severe energy crisis and the greenhouse effect.^[^ ^1^ ^]^ Currently, the widely used lithium‐ion batteries comprised of conventional graphite anodes and lithiated transition metal oxides cathodes have been proven to approach their property ceiling.^[^ ^2^, ^3^ ^]^ The expansion of lithium‐ion batteries with superior energy density and stability is expected to propose advanced electrode materials and new reaction theories, which, however, are always accompanied by challenging issues such as poor reversibility and stability because of the phase revolution and secondary reactions over electrochemical processes.^[^ ^4^, ^5^ ^]^ Of later years, metal–sulfur batteries (MSBs, M = Li, Na, K) have drawn significant attention and are recognized as potential future‐generation energy storage systems due to their overwhelming theoretical energy density, high specific capacity, and cost‐effective sulfur species.

Compared to lithium‐ion batteries, the MSBs include multistep electrochemical reactions, thus leading to high theoretic capacitance. However, the practical application of MSBs is still hampered by several critical challenges: i) the soluble polysulfides migrating to anodes, ii) the low conductivity of sulfur species, and iii) the sluggish sulfur redox kinetics, all of which result in poor sulfur utilization and inferior Coulombic efficiency. To meet these critical challenges, the ideal microstructure construction for sulfur host electrodes with intensifying the trapping and conversion of polysulfide molecules is of dominant significance. Moreover, the sizes, hierarchical architectures, conductive networks, heteroatoms doping, and functional components of diverse sulfur host electrodes are of great consequence in the adjustment of batteries’ performances. Therefore, reasonably engineering and functionalizing the electrodes with well‐defined chemical structures and accurate compositions serve a crucial role in promoting the charging–discharging performances and future commercialization of MSBs, especially electrode nanostructures designed by the multifaceted functionalization at molecular and atomic levels.

Benefiting from the tunable chemical compositions, large porosities, high surface areas, and huge family members, the metal‐organic frameworks (MOFs) and MOF‐derived nanostructures have been recognized as a new class of promising and rapidly developing multifaceted materials for a broad range of applications in the past ten years, such as the catalysts, energy storage, environmental science, and other fields.^[^ ^6^, ^7^, ^8^ ^]^ When using pristine MOF for catalytic reactions, it not only possesses high‐density and easily assessable catalytic active sites but also exhibits uniform porous structure and numerous channels to promote the transport and diffusion of reactants and products. Additionally, the facile functionalization of MOFs provides excellent opportunities for synthesizing heterogeneous and highly catalytic active nanostructures, such as functional modification, pore confinement, encapsulation, doping heteroatoms, and thermal treatment. Particularly, the MOFs engineered electrode materials facilitate accurate construction and characterization of catalytic sites at the molecular/atomic level, contributing to clarifying the catalytic mechanisms and affording great perception into constructing superior charging–discharging performances. During the last decade, many efforts have been devoted to this emerging area, abundant high‐performance and long‐life MSBs have been achieved. However, the recent progress regarding the MOFs and MOF‐derived electrode materials in MSBs with fast kinetics and satisfied stability has not been well summarized and concluded.

Here, in this timely review, we presented the most recent advancements in designing MOF‐derived electrodes, including fabricating strategies, composition management, topography control, and electrochemical performance assessment. This review will focus on the recent remarkable progress of MOF‐engineered multifaceted electrodes with diverse nanostructures, such as the pristine/functionalized MOFs, conductive substance‐hybridized MOFs, and MOF‐derived porous carbon with metal compounds, single‐atoms, and catalytic heterostructures. Particularly, we systematically discussed the inherent charge transfer, intrinsic polysulfide immobilization, and catalytic conversion on designing and engineering of MOF nanostructures for efficient MSBs. In the end, we expose the essence of how MOFs’ nanostructures influence their electrochemical properties in MSBs and conclude the future tendencies regarding the construction of MOFs and MOF‐derived electrodes in MSBs. We believe that this progress review will provide experimental/theoretical guidance in designing and understanding the MOF‐derived nanostructures as multifaceted electrodes, thus offering promising orientations for the future development of fast‐kinetic and robust MSBs in broad energy fields.

## 2 Current Status and Limitations of MSBs

**Scheme 1:**
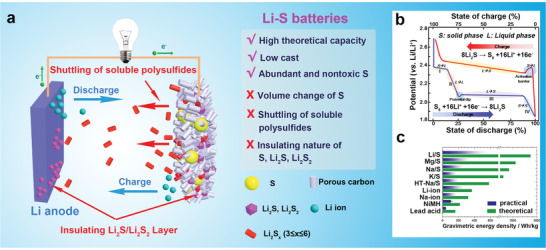
a) Schematic illustrations of the merits and issues in LSBs. b) The typical charge‐discharge CV plot of Li‐S chemistry. Reproduced with permission.^[^ ^13^ ^]^ Copyright 2019, Wiley‐VCH GmbH. c) Cartogram of gravimetric energy densities of current conventional MSBs systems. Reproduced with permission.^[^ ^11^ ^]^ Copyright 2017, Springer International Publishing AG.

Rechargeable MSBs have attracted extensive concerns mainly because of theoretical high energy density, high abundance of elements, and low cost. Nevertheless, even though decades of investigation, they are still reserving deficiency in long‐term lives, round‐trip efficiency, and sustainability. For example, the lithium–sulfur batteries (LSBs), among the state‐of‐the‐art renewable energy storage technologies, are considered one of the anticipated candidates for MSBs to meet the commercialization requirements. LSB arises an overwhelming theoretic gravimetric energy density (2600 Wh kg^−1^), which is five times as high as that of well‐used lithium‐ion batteries (**Scheme** **1**).^[^ ^9^, ^10^, ^11^ ^]^ Generally, LSBs present a multiple‐electron transfer procedure (S_8_ → Li_2_S_8_/Li_2_S_6_ → Li_2_S_4_ → Li_2_S_2_/Li_2_S), which results in high theoretic capacitance of sulfur.^[^ ^12^, ^13^ ^]^ However, these multistep reactions also bring problems and challenges, which seriously encumbered their commercialization process: i) the poor sulfur utilization due to the insulation nature of sulfur species; ii) the solubility of polysulfide molecules generating an internal “shuttle” behavior, leading to reduced coulombic efficiency and fast capacity decline; iii) the redox kinetics being more depressed owing to the rareness of active surfaces and insulation of sulfur cathode, resulting in low sulfur utilization and inferior charge efficiency; iv) the enormous volume change (80%) during cycling.

Compared with the flourishing LSBs, other types of MSBs, such as potassium–sulfur batteries (KSBs) or sodium–sulfur batteries (NSBs) participate in several same issues, mainly in regards to the stability of sulfur as well as the migration of polysulfides. Similar to LSBs, the generated polysulfide intermediates in Na–S and K–S systems tend to be too soluble in specific organic liquid electrolytes, which gives rise to the terrible shuttle phenomenon during cycling. Besides, the significant volume expansion and poor conductive of S species may result in instability and low electron transfer of electrodes. Therefore, the redox reaction kinetics and utilization of the superior capacity of S species in NSBs and KSBs get extremely depressed, leading to large battery polarization.^[^ ^14^, ^15^ ^]^

To address the above‐mentioned challenges in MSB system, a key point is to explore appropriate sulfur host materials with suitable chemical (polarity), physical (high specific area and conductivity), and electrochemical (exposed catalytic sites and surfaces) properties, as well as unique morphology (hierarchical, hollow, or core–shell architectures), which can offer not only strong polysulfides confinement from their bifunctional physicochemical adsorption sites but also ensure fast redox kinetics with their multiple open catalytic centers.

**Scheme 2:**
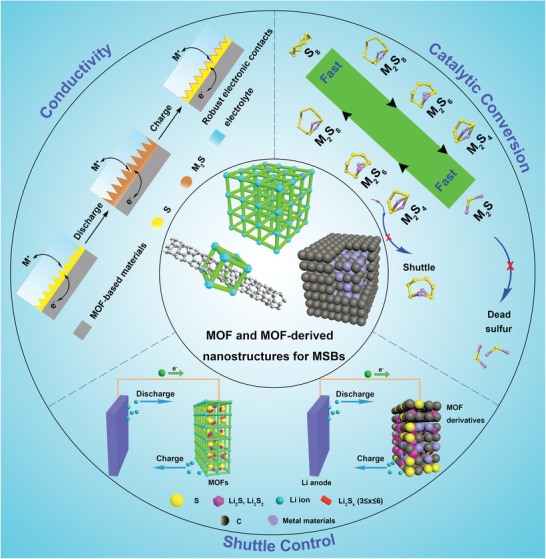
Illustrative picture for the advantages of MOF‐derived electrode nanostructures for MSBs.

As a new family of porous materials, MOFs constructed by coordinating metal ions/clusters with organic likers exhibit high crystallinity and ordered array. Considering their fascinating features of high porosity, large specific surface area, tunable topological structures, and adjustable chemical ingredients, MOF and MOF‐derived nanostructures have been demonstrated highly competitive to other common porous materials in MSBs.^[^ ^16^, ^17^ ^]^ As for the essential behaviors of sulfur cathodes, diverse types of MOF and MOF‐derived nanostructures play multifaceted roles in serving as electrodes for MSBs (**Scheme** **2**).^[^ ^18^ ^]^ i) Open metal sites and polar functional groups in MOFs contribute to powerful chemical affinity with polysulfides. ii) Uniform porous structures, tunable aperture led to effective polysulfides confinement and easy diffusion of electrons and reactants. iii) Heteroatom doping exhibits efficient chemical adsorbing polysulfides via polar surfaces. iv) Nanostructured MOFs provide significant advantages to realize high S loading and fast mass transfer. v) Conductive MOFs/MOF hybrids are expected to reduce internal resistance and promote fast electron transport. Moreover, vi) MOF‐derived metal compounds, single‐atoms catalysts, and catalytic heterostructures offer highly effective active centers and catalytic surfaces to accelerate polysulfides redox kinetics.^[^ ^19^, ^20^, ^21^ ^]^ Here, a comprehensive review on the structure, composition, and morphology design, as well as the electrochemical property, reaction theory, and practical use is noticeable, which can offer strategic guidance and overwhelming inspiration for the ingenious design and oriented development of MOF‐based MSBs and their commercialization in energy‐related applications.

In the following section of this review, we will summarize the recent research progress of MOF‐derived nanostructures for multifaceted electrodes in MSBs. Current breakthroughs in fabricating MOF electrodes with diverse nanostructures are systematically summarized, such as the pristine/functionalized MOFs, conductive substance‐hybridized MOFs, and MOF‐derived porous carbon with metal compounds, single‐atoms, and catalytic heterostructures. Particularly, we discussed the inherent charge transfer, intrinsic polysulfide immobilization, and catalytic conversion on designing and engineering of MOF nanostructures for efficient MSBs in depth.

## 3 Shuttle Control by MOF‐Derived Nanostructures

### 3.1 Pristine MOFs Nanostructures

MOFs as porous multifunctional materials engineered with inorganic metal sites and organic linkers have been widely studied in diverse battery systems in the past few years. Their tunable porous channels and controllable chemical components at the molecular and atomic levels have proved to be beneficial for improving the performance of MSBs. **Table** **1** lists some recent studies about MOF‐related materials for MSBs.

**Table 1:** MOF and MOF‐derived nanostructures for MSBs. (UHCS: ultrahydrophilic carbon stacks, LPS: lithium thiophosphate, Benz: benzoic acid, BTC: 1,3,5‐benzenetricarboxylic acid, GO: graphene oxide, TCPP: 5, 10, 15, 20‐tetrakis (4‐carboxyphenyl) porphyrin, PymS: 2‐mercaptopyrimidine, HITP: 2,3,6,7,10,11‐hexaiminotriphenylene, CNT: carbon nanotube, PPY: polypyrrole, CIMC: chemically integrated monolithic carbon, ZDC: ZIF derived carbon, NGC: N‐doped graphitic carbon, NCNF: N‐doped carbon nanofiber, NCNF: nitrogen‐doped carbon nanotubes, HNCP: hollow nitrogen‐doped carbon, NPC: nitrogen‐doped porous carbon, NGCNs: nitrogen‐doped porous carbon nanoshell, NPPCFs: N,P‐codoped porous carbon frameworks)

MOFs	Sample	Current density [C]	Capacity [mAh g^–1^]	Cycle	Potential[V]	Refs.
Pristine MOFs						
ZIF‐8	S/ZIF‐8	0.1	710	60	1.8–2.8	^[^ ^22^ ^]^
[Cu_2_Cl_2_C_10_H_8_N_2_]*_n_*	UHCS‐900 °C	1.0	≈580	100	1.8–2.8	^[^ ^23^ ^]^
UiO‐66	LPS‐UiO‐66(50Benz)	1.0	375	600	1.6–2.9	^[^ ^24^ ^]^
Cu_3_(BTC)_2_	Cu_3_(BTC)_2_@GO	0.2	1072	1500	1.5–3.0	^[^ ^25^ ^]^
MIL‐100(V)	MIL‐100(V)/rGO	0.1	879	200	1.6–3.0	^[^ ^26^ ^]^
Cu_2_(CuTCPP)	Cu_2_(CuTCPP)	0.2	953	900	1.6–2.8	^[^ ^27^ ^]^
Ni_2_(PymS)_4_	Ni‐MOF‐2D	0.1	516	1000	1.6–2.4	^[^ ^28^ ^]^
Ni_3_(HITP)_2_	Ni_3_(HITP)_2_/PP	0.2	1244	500	1.7–2.8	^[^ ^29^ ^]^
Ce‐MOFs	Ce‐MOF‐2/CNT	0.2	1140.7	800	1.7–2.8	^[^ ^30^ ^]^
MOFs composites						
MIL‐53	ppy‐S‐in‐MIL‐53	0.5	900	100	1.8–2.7	^[^ ^31^ ^]^
MOF‐5	MOF‐5@CNTs	0.5	540	50	1.5–3.0	^[^ ^32^ ^]^
HKUST‐1	HKUST1‐CIMC	0.2	757	300	1.7–2.8	^[^ ^18^ ^]^
ZIF‐8	ZDC@ZIF‐8	0.2	1410	300	1.6–2.8	^[^ ^33^ ^]^
MOFs derivatives						
ZIF‐67	Co‐NGC@NCNF	0.5	1007	500	1.7–2.8	^[^ ^34^ ^]^
MOF‐Celgard	Co_9_S_8_‐Celgard	0.1	1385	1500	1.8–2.8	^[^ ^35^ ^]^
Co‐MOF	CP@NCNT@CoS_3_	0.1	1228	400	1.7–2.8	^[^ ^36^ ^]^
ZIF‐8	Li_2_S‐ZnS@NC	0.2	832	1000	1.7–2.8	^[^ ^37^ ^]^
ZIF‐8	HNPC‐900‐65S	0.05	1605	500	1.6–2.8	^[^ ^38^ ^]^
ZIF‐67	CC@CoP/C‐S	0.1	1589	600	1.7–2.5	^[^ ^39^ ^]^
ZIF‐8/ZIF‐67	S‐NPC/G	1.0	608	300	1.7–2.8	^[^ ^40^ ^]^
ZIF‐67	CoS_2_@NGCNs	0.1	900.3	100	1.5–3.0	^[^ ^41^ ^]^
NPMOF	NPPCFs	0.5	840	200	1.7–2.8	^[^ ^42^ ^]^

#### 3.1.1 Structural Designs

**Figure 1:**
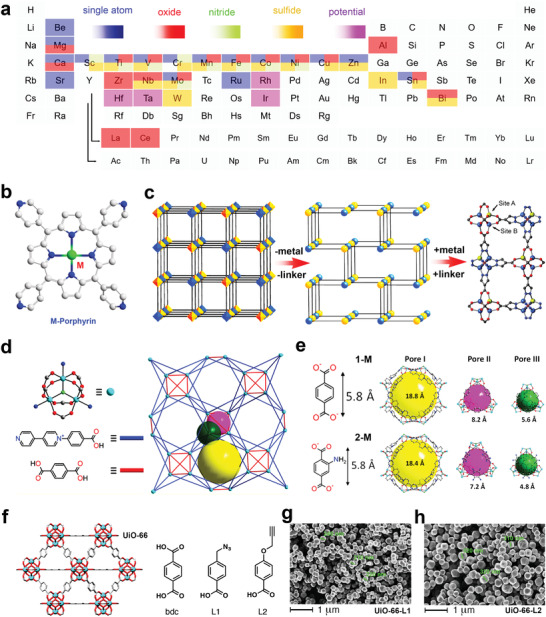
a) Schematic illustrations of reported and potential nanomaterials for MSBs. b) Metal‐porphyrin organic ligand. c) The metals and linkers removing reactions and other metals and new linkers insertion reactions in Zn_4_O(PyC)_3_. Reproduced with permission.^[^ ^53^ ^]^ Copyright 2014, American Chemical Society. d) The coordination and e) pore structures of zwitterionic MOFs. d‐e) Reproduced with permission.^[^ ^55^ ^]^ Copyright 2018, American Chemical Society. f) Uio‐66 with different linkers. g) The particle size of UiO‐6 with L1. h) The particle size of UiO‐6 with L2. f–h) Reproduced with permission.^[^ ^60^ ^]^ Copyright 2017, Elsevier Inc.

Until now, various nanomaterials have been reported as cathode hosts in MSBs (**Figure** **1**a), including single atoms (Be, Mg, Ca, Sr, Kc, Ti, V, etc.), transition metals (Mo, Cr, Mn, Cu, Ni, etc.) oxides, nitrides, and sulfides. Besides, metal nanoparticles (Pt, Co, Ni, etc.), metal carbides (W, Mo, Ti, etc.), and metal hydroxides (Fe, Co, Mg, etc.)^[^ ^43^ ^]^ also have been investigated as S hosts in MSBs. It is worth mentioning that nanomaterials containing V, Cr, Ln, and Ce show outstanding electrochemical performance among all reported materials.^[^ ^44^, ^45^, ^46^ ^]^ Although most of the metal elements have been applied to study the mechanism in MSBs, there is still some potential metal, such as Hf, Ta, Rh, etc. that possess similar electronic configuration to the outstanding ones, which can be beneficial to achieve high capacity and stability. Therefore, it is necessary to design desired cathode hosts, such as MOFs, to make the most use of these potential metals.

Benefiting from the high specific area, tunable porous structures, and a variety of metal nodes and organic ligands, MOFs provide extensive opportunities to produce advanced cathodematerials with improved coulombic efficiency and cycling stability in MSBs.^[^ ^25^ ^]^ Notably, the accessible metal sites and polar functional groups locating on their organic ligands can be created into the array of MOFs, both of which can bond robustly with soluble polysulfides. Furthermore, the large specific area can result in high sulfur loading, while the aperture dimension of the pores can physically restrict the mobile polysulfides. Therefore, designing MOFs with desired functionalities is primary, including suitable pore structure, adjustable particle size, and accessible Lewis acid/base sites.

Generally, the presynthetic design strategies or postsynthetic modification can be taken to synthesize MOFs with open metal centers. For example, metalloporphyrins can offer great potential to build blocks for the construction of stable MOFs with accessible metal sites via pre‐synthetic design (Figure 1b). The Hupp group described a successful preparation of an expanding family of MOFs that could directly involve varieties of metalloporphyrins (such as Al^3+^, Zn^2+^, Pd^2+^, Fe^3+^, and Mn^3+^ metalloporphyrins).^[^ ^47^ ^]^ Remarkably, these stable porphyrinic materials featured large cavities and lightly accessible active nodes, leading to metal‐site‐based applications. Besides, another idea of double metal sites’ exposure through post‐synthetic modification in MOFs may provide potential advantages in polysulfide confinement.^[^ ^48^, ^49^, ^50^, ^51^, ^52^ ^]^ The removal and interpolation of both ligands and metal ions inside the array of MOF‐5 analog of [Zn_4_O(PyC)_3_] (PyC = 4‐pyrazo lecarboxylate) have been investigated,^[^ ^53^ ^]^ in which ordered defects can be generated (Figure 1c). When added into water, one‐half of the ligands and a quarter of metal ions could be removed in a moment. The producing crystal possessed regular metal and linker vacancies, with complete preservation of crystallinity.

Because the small apertures in an ordered framework possess an affinity with the polysulfide molecules, while big mesopores can contribute to electrons/mass transfer.^[^ ^54^ ^]^ Therefore, it is feasible to design hierarchical porous structures to help achieve high capacity and stable cycling. A sequence of porous zwitterionic MOFs has been investigated by introducing varieties of ditopic carboxylate ligands with abundant functional groups (Figure 1d).^[^ ^55^ ^]^ The pore system of zwitterionic MOFs featured three categories of pores being scientifically adjusted in dimension scope from 17.4 to 18.8 Å (named pore I), 8.2 to 12.8 Å (named pore II), and 4.8 to 10.4 Å (named pore III) via the utilization of auxiliary ligands (Figure 1e). Meanwhile, these pore surfaces were arranged with charge gradients formed by employing the pyridinium ligands, which possessed polarization effects. As a result, through this novel design, it may achieve large S loading and fast ion/electron transform by pore I and pore II. Besides, four similar Ni‐MOF‐74 have been designed by adjusting the number of phenylene rings and the relative positions of hydroxyl‐ and carboxylate.^[^ ^56^ ^]^ Interestingly, these MOFs show a controllable range of pore sizes of 11 Å → 27 Å; the presented hierarchical pores may realize satisfactory S loading and excellent confinement effect towards polysulfides.

It has been proved that suitable particle size of MOFs (100–200 nm for ZIF‐8) was favorable for achieving a high capacity in MSBs,^[^ ^57^ ^]^ in which too small size leading to leaching of soluble polysulfides, while larger size lowering the mass and electron transport. Therefore, synthesizing MOFs with the particle sizes of 100–200 nm could be maximizing sulfur utilization and increasing rate capabilities. According to the theory of nucleation and growth,^[^ ^58^ ^]^ the short nucleation period is pivotal for preparing homogeneous nanoparticles. To acquire small and homogeneous MOF particles, it is primary to create many nuclei through burst nucleation, subsequently to abort crystal growth via consumption of all ingredients rapidly. Hence, most MOF NPs with nanoscale sizes were prepared via rapid nucleation or coordination modulation.

For example, in an integrated rapid nucleation research of Fe(OH)BDC (BDC = terephthalate) nanoparticle crystallization,^[^ ^59^ ^]^ microwave, ultrasound, and typical electroheat approaches were adopted to reveal the influence of nucleation rate and growth kinetics of MOFs. Results disclosed that the crystal growth rate was equivalent over all three approaches; however, MOF nanoparticle sizes (heating >> microwave > ultrasound) are usually negatively related to their nucleation rates. Besides, the size and morphology of MOF nanoparticles can be adjusted by bringing in modulators, such as monotopic benzoic acid, assisting their preparation. A key example of coordination modulation synthesis has been studied by a renewable two‐step strategy, termed “click modulation,” where the functionalized modulators on MOFs’ external surfaces can effectively regulate particle sizes and then further modify them through postsynthetic regulation.^[^ ^60^ ^]^ Here, *p*‐azidomethylbenzoic (named L1) and *p*‐propargyloxybenzoic (named L2) were chosen as click modulators to synthesize UiO‐66 in which UiO‐66‐L1 possessed a uniform nanoparticle size of 100–200 nm, while UiO‐66‐L2 exhibited apparent larger particle sizes (400–600 nm) (Figure 1f–h), meaning that click modulation could be well applied to regulate the sizes of MOFs.

#### 3.1.2 Applications in MSBs

Since the pioneering reports of MOF structures designed by the Yaghi group,^[^ ^61^ ^]^ Fe‐MOFs were subsequently employed as the electrode material in lithium‐ion batteries,^[^ ^62^ ^]^ and various types of MOFs have been searched for battery‐related utilizations. Meanwhile, when applied as a host matrix in MSBs, the metal nodes and organic ligands of MOFs have exhibited strong chemical affinity with polysulfides, along with a highly extensive porosity as well as a large specific surface area that enable the accommodation of abundant sulfur.^[^ ^63^, ^64^ ^]^ Another significant feature should be their ability to restrain active functional guests and their tendency in producing highly adjustable nanostructures, all characteristics render MOFs potential sulfur hosts for MSBs.

MOFs equipped with diverse active sites can strongly bond with soluble polysulfides to effectively limit their dissolution.^[^ ^65^ ^]^ Generally, metal ions such as active transition element Ti^4+^, V^4+^, Mn^4+^, Co^2+^, and the rare species Nb^5+^, Ru^3+^, etc. are widely employed to construct the binding sites for the polysulfide (S*_x_* ^2–^) anion, while nonmetallic heteroatoms, such as B, N, O, F, and S, are usually applied to connect with the metal ion (Li^+^, Na^+^, K^+^) in MSBs.^[^ ^66^, ^67^, ^68^, ^69^, ^70^, ^71^ ^]^ For example, computation of 16 metal‐replaced isomorphisms of M_2_(dobdc) (dobdc = 2,5‐dioxido‐1,4‐benzene dicarboxylate) was carried out to study their capability to chemically immobilize sulfur species (such as S_8_, Li_2_S_4_, and Li_2_S, etc.) during the charge‐discharge process in LSBs, identifying that the Ti_2_(dobdc), Ni_2_(dobdc), and Mo_2_(dobdc) possess superior interactions with Li_2_S_4_.^[^ ^72^ ^]^

**Figure 2:**
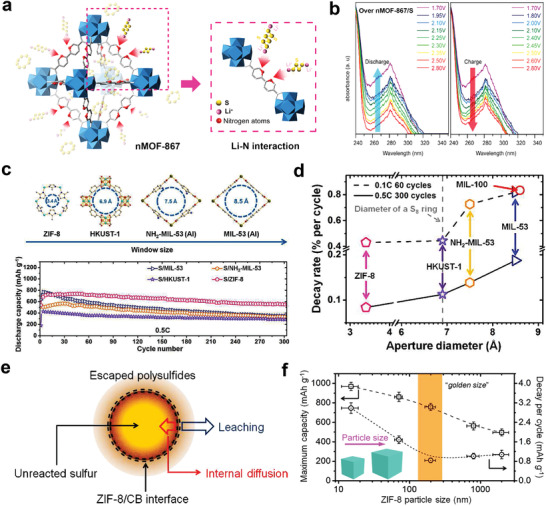
a) The sp^2^ nitrogen sites and lithium polysulfides connect chemically in nMOF‐867. b) The in situ infrared spectra of nMOF‐867 in LSB. a,b) Reproduced with permission.^[^ ^73^ ^]^ Copyright 2016, Nature Publishing Group. c) Cycling stability at 0.5 C and d) average recession rate of different pore sizes in MOFs. c,d) Reproduced with permission.^[^ ^22^ ^]^ Copyright 2014, The Royal Society of Chemistry. e) Schematic model of a S@ZIF‐8 particle in discharge and maximum capacities. f) The average recession per cycle. e,f) Reproduced with permission.^[^ ^57^ ^]^ Copyright 2015, The Royal Society of Chemistry.

On the other hand, the polar functional groups (Lewis basic sites) on their organic linkers can offer strong affinity abilities toward polysulfides and provide effective polysulfides confinement within MOF pores. Park and co‐workers hold the first direct example to explain the essence and combining lithium polysulfide capacity with polar heteroatoms modified on the ligands of MOFs.^[^ ^73^ ^]^ They employed the nanosized MOF‐867 as a cathode host with sp^2^ nitrogen as Lewis basic sites on its linkers (**Figure** **2**a). As a contrast, MOFs with the identical configurations as nMOF‐867 but without sp^2^ nitrogen sites on its linkers, named as nUiO‐67, was also acquired and discussed. It was found that the first discharge efficiency of these two MOFs remained nearly the same; however, after long period cycles, the remainder property of nMOF‐867 was better than that of nUiO‐67, which could be ascribed to the interaction between sp^2^ nitrogen sites with polysulfides. Meanwhile, the confinement effect of nMOF‐867 was confirmed by in situ spectroelectrochemical test (Figure 2b). The spectra showed an increased absorption intensity of nMOF‐867/S during the discharge process and an inverse change during charge reactions, which could be ascribed to the light scattering from the polysulfides contained inside the small cavities of nMOF‐867.

The pore structure of carbonaceous cathode has been demonstrated to play a critical role in MSBs.^[^ ^74^ ^]^ Generally, those possessing large pores can load more S while their open frameworks can scarcely limit the shuttle effect of mobile polysulfides; on the contrary, those with small pores can strongly suppress the shuttle effect of soluble polysulfides while the S storage and the electron/mass migration are restrained. MOFs have controllable pore structures with well‐defined cavities and channels constructed by the connection of metal ion/cluster with organic ligands. Therefore, the adjustable pore structures and high specific surface area of MOFs can support a great deal of sulfur inside their pores and constrain the mobile intermediate polysulfides. For example, a mesoporous MOF, MIL‐100(Cr), with two types of cage‐shape pores (≈2.4 nm and ≈2.9 nm) combined via two kinds of channels (≈0.5 nm and ≈0.9 nm) was studied as a sulfur cathode for LSB.^[^ ^75^ ^]^ Notably, 48 wt% of S loading could be loaded inside the pores of MIL‐100(Cr) by employing a melt‐diffusion method. The generated MIL‐100(Cr)/S composite materials showed outstandingly higher long‐cycling stability than the conventional porous carbon/S composites, which could be attributed to the advantages of the cage‐shape pore structures of MIL‐100(Cr) beyond typical porous carbon materials.

Furthermore, the battery properties of four types of MOFs with diverse pore sizes (i.e., 0.34 nm for ZIF‐8, 0.69 nm for MIL‐53(Al), 0.75 nm for NH_2_‐MIL‐53(Al), and 0.85 nm for HKUST‐1, respectively) were studied.^[^ ^22^ ^]^ It was demonstrated that the performance declining was directly related to their pore sizes for the four kinds of MOFs, and the average recession rate at 0.5 C were 0.08% for ZIF‐8, 0.11% for MIL‐53(Al), 0.14% for NH_2_‐MIL‐53(Al), and 0.19% for HKUST‐1 per cycle, respectively (Figure 2c,d), suggesting the critical roles of the pore sizes on the anchor of soluble polysulfides thereby promoting cycling stability.

The particle sizes of MOFs also play a vital role in MSBs properties. Just like Li‐ion batteries, smaller particle sizes can be beneficial for the mass/electron transport and can produce an easier contact with the liquid electrolyte and a conductive agent. For the MSBs, nevertheless, the fleeing of polysulfides can be induced with the decreasing of particle sizes, which causes situations more severe.^[^ ^76^ ^]^ Given this circumstance, Li and co‐workers comprehensively researched the effect of the particle sizes of MOFs when serving as cathode materials in LSBs.^[^ ^57^ ^]^ A series of ZIF‐8 particles with continuously varying dimensions (i.e., 15 nm → 70 nm → 200 nm → 800 nm → 2000 nm) were controllably prepared and applied as S host materials. It was demonstrated that the coulombic efficiency and sulfur utilization fell off along with the rising of particle sizes; however, the performance of ZIF‐8 with smaller sizes declined more quickly. A mechanism was raised to demonstrate the vital phenomenon of the specific “golden size” (Figure 2e,f). When ZIF‐8 particles with sizes larger than a definite demarcation (≈200 nm), a longer time need to be consumed for polysulfides diffusing inside and interacting with the S species inside ZIF‐8 pores, leading to the inferior usage rate of sulfur; on the other hand, as for ZIF‐8 with a smaller size, the specific surface area was too large to the polysulfides, which could easily flee from the ZIF‐8 support, thus resulting in unstable cycling. Consequently, it is crucial to consider both S utilization and long‐term life for choosing appropriate MOF sizes to create superior sulfur hosts in MSBs.

### 3.2 MOF‐Derived Hybrids

#### 3.2.1 Structural Designs

Heteroatom doping or constructing strategic topographies of MOFs derivatives are effective strategies to hinder polysulfides’ dissolution and improve utilization of active sulfur in MSBs. Herein, heteroatoms, especially N, O, F, doping in porous carbon frameworks is in favor of achieving strong physical and chemical adsorption ability for polysulfides through the surface polarity and porous structures.^[^ ^77^, ^78^, ^79^, ^80^, ^81^, ^82^ ^]^ Besides, hollow carbon materials, especially those with double or even multilayered shells, are of great promise to realize high S loading and fast mass transfer by their large internal void space.^[^ ^83^ ^]^ Significantly, hierarchical porous structures of MOFs derivatives show similar properties with that of pristine MOFs, where small micropores promote confining polysulfides, while mesopores are beneficial to electrons/ions transfer. Therefore, synthesizing heteroatoms‐doped hierarchical porous or hollow carbon hybrids could be farsighted strategies for advanced MSBs.

MOFs with hierarchical pores can deliver a promising possibility in MSBs, where the ultramicropores will offer a strong confinement effect, while the larger mesopores are beneficial for promoting accelerated mass diffusion.^[^ ^84^ ^]^ Moreover, heteroatom doping in materials is considered a simple and efficient method to boost their chemical affinity to polysulfides. Therefore, the combination of heteroatom doping with micro‐meso‐ or micro‐meso‐macroporous materials could optimize their physical and chemical co‐confinement. For this purpose, a new type of hierarchically microporous and mesoporous N‐doped carbon nanosheet was investigated.^[^ ^85^ ^]^ Here, a polymer–polymer interfacial self‐building strategy was proposed, which offered an easy and effective method to create hierarchically porous N‐doped carbon nanosheets. Meanwhile, it also enabled synchronous and independent regulation of the thickness (5.6–75 nm), micropore sizes (0.6–1.4 nm), and mesopore sizes (25–46 nm).

**Figure 3:**
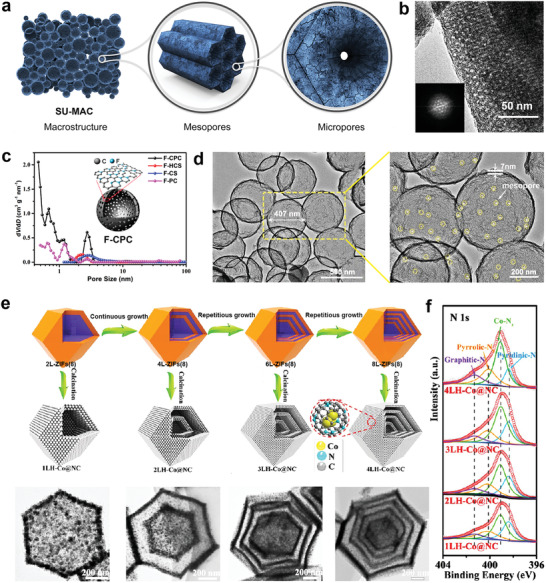
a) The hierarchical porous architectures and b) the TEM of the synthesized SU‐MAC materials. a,b) Reproduced with permission.^[^ ^84^ ^]^ Copyright 2016, American Chemical Society. c) The pore size and d) the fluorine‐doped cage‐like porous carbon (F‐CPC). c,d) Reproduced with permission.^[^ ^89^ ^]^ Copyright 2020, American Chemical Society. e) The structure of multishell‐ZIF‐8 and the derived hollow Co@NC polyhedrons with adjustable shell numbers. f) The XPS of hollow Co@NC polyhedrons. e,f) Reproduced with permission.^[^ ^94^ ^]^ Copyright 2019, American Chemical Society.

Significantly, an N‐doped trimodal porous structure, i.e., micro‐meso‐macroporous structure, has been studied by the Bao group. (**Figure** **3**a,b).^[^ ^84^ ^]^ They proposed a controlled preparation of nitrogen‐doped hierarchical carbon (SU‐MAC), where the macroporous structures were constituted via adjusting the electrostatic interaction. In contrast, the mesopores constituted through separating the soft triblock copolymer templates, and the ultra‐micropores (*d* < 0.5 nm) coming from the fragmentation of carboxylic groups and mutual diffusion of the triblock copolymer in polypyrrole. As a result, the production has hierarchical porous structures possessing macro‐, meso‐, and micropores and preserves nitrogen moieties. Moreover, of course, heteroatoms, such as (N, P),^[^ ^86^ ^]^ (N, P, S),^[^ ^87^ ^]^ or (N, P, O, B),^[^ ^88^ ^]^ etc., codoped hierarchical porous carbon should also serve as promising structures.

Hollow structures with inferior specific densities, high porosity, and larger specific surface areas can effectively abridge the diffusion path of electrons/ions, improve the electrode/electrolyte interphase, and offer accessional cavities to weaken the frequently existing volume varieties during cycling.^[^ ^89^, ^90^, ^91^, ^92^ ^]^ Hence, developing potential hollow carbon materials, especially the double‐ or multilayered‐shelled hollow materials combined with heteroatom doping or hierarchical pores, could provide a sufficient shuttle‐controlled effect.^[^ ^93^ ^]^ Recently, the Yan group proposed an F‐doped cage‐shape hollow hierarchical porous carbon (F‐CPC) by intentionally adjusting its structural performance (Figure 3c,d).^[^ ^89^ ^]^ The superior F‐CPC possessed large specific surface areas with robust mesopores, plenty of micropores, and fast charge transport. Lately, a series of hollow multishell dodecahedrons that possessed accurately regulated nanostructures and adjustable components were investigated.^[^ ^94^ ^]^ This novel solid‐to‐hollow evolution technique in the carbonization process, as well as the physicochemical controllability of ZIF species, offered possibilities to accurately regulate the shell numbers and metal components in the multishell hollow dodecahedrons (Figure 3e,f). Moreover, this method could also be applied to the building of varieties of core–shell and core–multishell hollow architectures.

#### 3.2.2 Applications in MSBs

Plenty of researches have demonstrated that MOFs are a new class of porous materials as the cathode in MSBs, while their charge transport needs further accelerated. Therefore, the concentrated studies of MOF‐derived materials have induced many considerations owing to their unexpected property as S supports in MSBs.^[^ ^95^ ^]^ Generally, the main advantages associated with shuttle control by MOFs‐derived materials can be attributed to two factors. i) Heteroatoms (N, P, S, etc.) doped porous carbon materials offer both strong chemical and physical affinity toward polysulfides.^[^ ^96^ ^]^ ii) Predesigned morphology, ultrahigh surface area, and hierarchical pores of precursor MOFs can be retained after high‐temperature pyrolysis,^[^ ^97^ ^]^ which can achieve high S loading and excellent physical polysulfides confinement. Notably, some other unique materials will be discussed in later sections, such as the MOF‐derived metal compounds, single‐atom catalysts, catalytic heterojunctions, etc.^[^ ^13^ ^]^ that exhibit both confinement and catalytic effect in MSBs.

**Figure 4:**
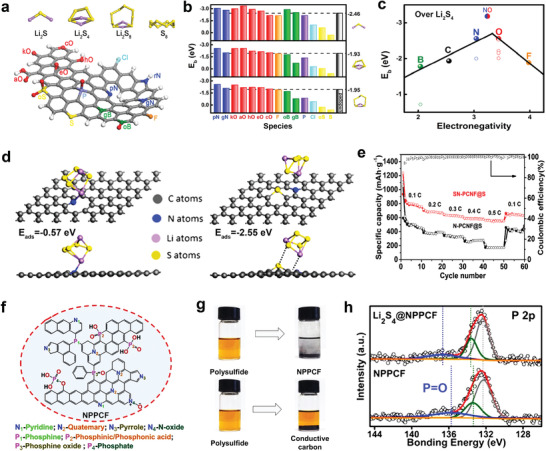
a) The configuration of polysulfides and X‐doped GNRs. b) The binding energy of polysulfides when interacting with X‐doped GNRs. c) The volcano figure of binding energy versus electronegativity of X‐doped GNRs. a–c) Reproduced with permission.^[^ ^99^ ^]^ Copyright 2016, Wiley‐VCH GmbH. d) Li_2_S_4_ adsorption on sulfur and nitrogen co‐doped porous carbon nanofibers (SN‐PCNF) and e) its cycle performance. Reproduced with permission.^[^ ^20^ ^]^ Copyright 2020, Elsevier B.V. f) The structure of N‐ and P‐containing carbon frameworks (NPPCFs). g) The ability of NPPCFs confining polysulfides. h) The XPS of NPPCF and Li_2_S_4_@NPPCF. f–h) Reproduced with permission.^[^ ^42^ ^]^ Copyright 2019, American Chemical Society.

Heteroatom (N, P, B, or S, etc.) doping in MOF‐derived porous carbon matrixes is a valid strategy to enhance physical and chemical adsorption toward polysulfides owing to the adjustment for their surface polarity and porous structures.^[^ ^98^ ^]^ For example, the Zhang group demonstrated that among all heteroatoms (N, O, B, F, S, P, etc.), nitrogen and oxygen dopant could markedly enhance the binding abilities of the carbon hosts with the polysulfide molecules in LSBs, where, more significantly, N, O codoping offered strongest polysulfides affinity (**Figure** **4**a–c).^[^ ^99^ ^]^ DFT calculation proved that to accomplish the robust confinement toward polysulfides, the criterion for the reasonable construction of doped porous carbon frameworks in an LSB depended on: i) the heteroatoms had lone pair electrons; ii) the heteroatoms exhibited higher electronegativities than carbon frameworks and appropriate radius that match Li atom; iii) the heteroatoms could form delocalized π‐bond interactions with the conjugated systems; iv) the heteroatoms could form robust bonds with the carbon plane.

Multiheteroatom codoped carbon materials usually exhibit stronger polysulfides confinement than that of monoheteroatom doped materials.^[^ ^100^ ^]^ Until now, plenty of heteroatoms codoped carbon materials have been employed as cathode hosts in MSBs.^[^ ^101^, ^102^, ^103^ ^]^ For example, the S and N codoped method was studied to improve the interactions between lithium sulfides with their hosts.^[^ ^20^ ^]^ It is proved that stronger polysulfide confinement could be realized by N and S codoped materials rather than those with undoped or N single‐doped materials due to the dual binding energy (Figure 4d,e). Meanwhile, the Voget group also revealed that high‐loaded heteroatom concentration (≈40 at%) of N and S could effectively trap the sodium polysulfides in NSBs,^[^ ^104^ ^]^ in which the electrostatic effect between the pore walls with S and the Na—N bonds could be responsible. Moreover, N, P‐codoped carbon skeletons were also realized in LSBs.^[^ ^42^ ^]^ Herein, N and P heteroatoms were involved on the surfaces of the porous carbon skeletons with a great deal of accessible polar groups (Figure 4f). Importantly, upon confining toward Li_2_S_4_, the P 2p XPS exhibited obvious positive moving (135.5 eV → 136.7 eV) at the peak of the P=O bond, suggesting that the oxophilie P=O groups could afford a strong binding ability with polar polysulfides. As a result, the highly polar surface, interconnected pores, and high specific surface area maximized the affinity towards polysulfide intermediations, resulting in high coulombic efficiency and superior stability (Figure 4g,h).

**Figure 5:**
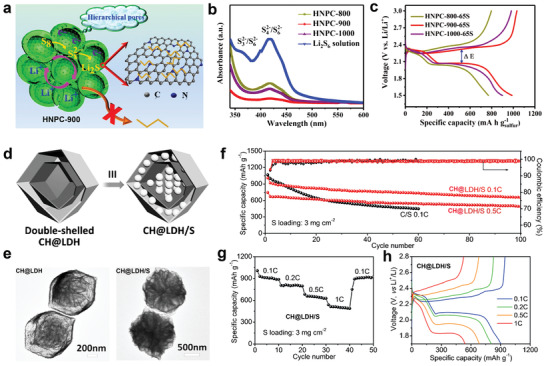
a) The HNPC structures. b) The confinement of HNPC toward polysulfides. c) The charge–discharge performances of HNCP in LSBs. a–c) Reproduced with permission.^[^ ^38^ ^]^ Copyright 2019, Wiley‐VCH GmbH. d) The schematic and e) SEM of dual‐layered CH@LDH cages. f) The cycle performance and g) the rate and h) charge–discharge performances of CH@LDH/S in an LSB. d–h) Reproduced with permission.^[^ ^109^ ^]^ Copyright 2016, Wiley‐VCH GmbH.

MOF‐derived hollow nanostructures, especially heteroatom‐doped hollow carbon nanostructures, as attractive architectures that received particular attention because of their excellent properties, such as high specific surface areas, short diffusion path, and fast mass‐migrating kinetics.^[^ ^14^, ^105^, ^106^ ^]^ Meantime, their considerable internal cavity space can contain comparatively high S ratio and stand great volume variety, while outer polar carbon shells can use as the cages to suppress the shuttle phenomenon via physical or chemical confinement. For example, synthesizing the interlaced porous carbon hollow nanospheres to serve as a significant matrix for NSBs.^[^ ^107^ ^]^ The successive and interconnected C backbone assured high tap densities and structural intimacy, while the internal void spaces could contain a high S loading and tolerate large volume expansion, thereby significantly enhancing electrochemical performance. Besides, a comparison of the performance difference between heteroatom doping and hollow carbon materials in LSBs has been studied.^[^ ^38^ ^]^ Through systematically testing and comparing the electrochemistry performance of mixed hollow N‐doped carbons (HNPC) (**Figure** **5**a–c), the solid N‐doped and N, S‐codoped carbons, where HNPC held superior performance mainly attributed to the synergistically enhanced architectures allowing for fast electron transport as well as the hollow structure being favorable for mass transfer.

Core–shell nanostructure can maximize the S loading and inhibit polysulfides dissolution through placing polar or porous media in the hollow interior, which settles the problems of the absence of strong adsorption sites in the interior of hollow architectures.^[^ ^108^ ^]^ For example, the Lou group proposed and prepared a new kind of dual‐layered Co(OH)_2_/LDH (denoted CH@LDH) nanocages to serve as a novel cathode material in LSBs (Figure 5d,e).^[^ ^109^ ^]^ With this design, the external LDH layers and internal Co(OH)_2_ hollow cores can maximize hollow architectures’ superiority for loading a great deal of S and provided comparatively large polar surfaces for chemically interacting with polysulfide molecules to restrain their migration (Figure 5f–h). Moreover, the electrochemistry performance of yolk–shell interconnected carbon nanospheres with hollow cavities has been investigated.^[^ ^110^ ^]^ A novel class of hollow yolk‐shell carbon spheres has been proposed, which consists of mesoporous carbon shells, hollow cavities, and microporous cores locally immobilized on their shells. Herein, the microporous carbon cores can decrease the inner electron transfer resistance and anchor the mobile polysulfide molecules, thereby facilitating a robust cycle property.

## 4 Enhancing the Conductivity of MOF‐derived Nanostructures

### 4.1 Pristine MOF‐Derived Nanostructures

#### 4.1.1 Structural Designs

**Figure 6:**
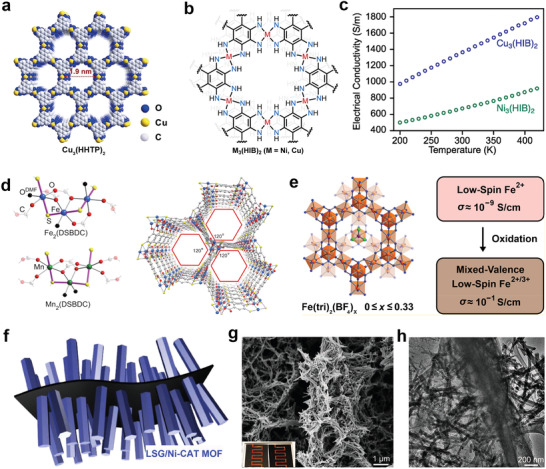
a) Conductive Cu_3_(HHTP)_2_. Reproduced with permission.^[^ ^114^ ^]^ Copyright 2020, Wiley‐VCH GmbH. b) The structure and c) the correlation between temperature and electrical conductivity in M_3_(HIB)_2_. b‐c) Reproduced with permission.^[^ ^115^ ^]^ Copyright 2017, American Chemical Society. d) The conductive M_2_(DEBDC) (M = Fe, Mn; E = S, O). Reproduced with permission.^[^ ^120^ ^]^ Copyright 2015, American Chemical Society. e) The conductive mixed‐valence Fe(tri)_2_(BF_4_)*_x_*. Reproduced with permission.^[^ ^121^ ^]^ Copyright 2018, American Chemical Society. f) The schematic, g) SEM, and h) TEM of LSG/Ni‐CAT MOF. Reproduced with permission.^[^ ^138^ ^]^ Copyright 2019, Wiley‐VCH GmbH.

Designing conductive MOFs is an available method to overcome the S cathode's insulating nature and simultaneously maintain the porosity and specific area of MOFs from been destroyed. Generally, tracing a clue via the more developed field of conductive molecules or coordination polymers, it can be demonstrated that two essential strategies will be applied to design novel MOFs with excellent electrical conductivity.^[^ ^111^, ^112^, ^113^ ^]^ One is the so‐called “through‐space” strategy, which uses π‐stacks of conductive molecules as the charge transport pathways and features expanding 2D π‐conjugation and layer‐by‐layer architectures that are similar to graphene to offer high conductivity and large charge mobility. For example, Cu_3_(HHTP)_2_,^[^ ^114^ ^]^ that formed by linking the highly conjugated 2,3,6,7,10,11‐hexahydroxytriphenylene (HHTP) with Cu(II) ion to 2D porous expanding skeletons (**Figure** **6**a), exhibited superior electroconductivity and charge storage abilities due to the tight π‐stacking facilitating effective linker orbital overlap along with the crystallographic orientation. Moreover, M_3_(HIB)_2_, where M = Ni, Cu, with in‐plane π‐conjugation structure was realized.^[^ ^115^ ^]^ Here, the interaction of hexaaminobenzene (HIB) with Ni^2+^/Cu^2+^ through discreetly controlled conditions could generate porous crystal materials with excellent charge transfer properties (Figure 6b). Meantime, it was found that the electron transport of the synthesized Ni_3_(HIB)_2_ and Cu_3_(HIB)_2_ exhibited a positive correlation with the test temperature in which both MOFs exhibited high electrical conductivity of 0.8 S cm^−1^ in Ni_3_(HIB)_2_ and 1.3 S cm^−1^ for Cu_3_(HIB)_2_ at 300 K (Figure 6c).

Another approach to construct conductive MOFs depends on a “through‐bond” form, in which both symmetry and orbital overlap within the covalently combined compositions need to exist to facilitate fast charge conveyance.^[^ ^116^, ^117^, ^118^ ^]^ This strategy is usually employing soft S‐based bridging ligands, such as di‐ or tetra‐thiobenzenes, which can produce more covalent bonding with metal ions.^[^ ^119^ ^]^ For example, the reported famous MOF‐74 analog “M_2_(DSBDC)” (M = Mn, Fe) (DSBDC = 2,5‐disulfhydrylbenzene‐1,4‐dicarboxylic) exhibited high charge transport due to the infinite metal‐sulfur chains (Figure 6d).^[^ ^120^ ^]^ Density functional calculations revealed that (—Fe—S—)_∞_ links in Fe_2_(DSBDC) could result in the fastest charge transport in the MOF‐74 analogs and higher electrical conductivity. Furthermore, it was found that the electrical conductivity increased remarkably with the Fe oxidation level.^[^ ^121^ ^]^

Coupling MOFs with other conductive materials has been recognized as another effective method to realize high electronic conductivity.^[^ ^122^, ^123^, ^124^, ^125^ ^]^ Hence, designing MOF composites together with desired conductive polymers or carbon is promising to construct ideal cathode frameworks and deliver a large discharge capacity.^[^ ^126^, ^127^ ^]^ Notably, the Cui group concluded that the conductivity of the common conductive polymers,^[^ ^128^ ^]^ like polyaniline (denote PANI), polypyrrole (denote PPY), and poly(3,4‐ethylenedioxythiophene) (denote PEDOT), increased as follows: PANI < PPY < PEDOT, suggesting that the PEDOT possess the best conductivity. Therefore, exploiting potential MOF composites with PEDOT could lead to high conductivity, thus promoting low internal resistance and fast electron migration. For example, Salcedo‐Abraira and co‐workers successfully accommodated PEDOT into the mesopores of MIL‐100(Fe) via in situ oxidative polymerization of EDOT monomer inside MOF pores.^[^ ^129^ ^]^ They found that the electroconductivity of the as‐synthesized PEDOT@MIL‐100(Fe) materials was enhanced compared with the isolated MIL‐100(Fe) and conductive PEDOT. Recently, the Ballav group prepared porous semiconductor composites via assembling PPy and PEDOT links into the cavities of UiO‐66.^[^ ^130^ ^]^ Remarkably, due to the essential double porosity in UiO‐66, polymer links were discovered to selectively settle down inside only one void. This led to an extraordinary improvement (million‐fold) of the electroconductivity while this composite could hold 60–70% porosity of its parent MOF.

MOF composites containing conductive carbon materials, such as GO, rGO, CNTs, etc.,^[^ ^131^, ^132^ ^]^ can also deliver high conductivity and be designed for high‐efficiency MSBs. Graphene‐based materials have recently attracted great attention owing to their particular architectures and outstanding physical and chemical performance.^[^ ^133^ ^]^ Based on current studies, composites of graphene/MOF‐based materials could integrate their superiorities and eliminate individual defections, leading the composites with enhanced stability and improved electroconductivity.^[^ ^134^, ^135^, ^136^ ^]^ Alkordi and co‐workers produced a composite of HKUST‐1/graphene by one‐pot synthesis technology.^[^ ^137^ ^]^ They found that the moldable composite materials allowed for an adjustable graphene content, in which the high electrical conductivity (from 7.6 × 10^–3^ to 640 S cm^−1^) was positively correlated to the graphene loadings. Besides, a laser scribing technique was proposed by the Alshareef group.^[^ ^138^ ^]^ Here, 1D conductive Ni‐catecholate‐based MOF (Ni‐CAT) was grown on a patterned 3D laser scribed graphene (LSG) electrode (Figure 6f–h). Notably, the LSG could serve as conductive 3D matrixes for selective and homogeneous depositing Ni‐CAT nanorods, thereby showing significant improvement in conductivity.

As a result, to make the best of the advantages of MOFs, improving the poor intrinsic properties of MOFs should be a useful development means in MSBs, such as the high porosity, designing conductive MOFs, or combining MOFs with high conductive materials. Thus, the electrochemical performances will be improved in the case of coulombic efficiency, rate property, and cycling stability as the conductivity of MOF materials is optimized.

#### 4.1.2 Applications in MSBs

The insulative feature of both S and the end discharge product M_2_S (M = Li, Na, K) can be a tremendous scientific challenge that hinders the commercialization of MSBs. As is well known, M_2_S is both electronic and ionic insulating. In general, for example, stoichiometric Li_2_S possesses a resistance higher than 10^14^ ohm cm, and the Li^+^ ion conductivity in Li_2_S is low to 10^–15^ cm^2^ s^−1^.^[^ ^139^ ^]^ Once the total electrode is entirely covered by a thin Li_2_S layer, further lithiation may be significantly hindered, and the potential declines quickly. Hence, the full conversion of S to Li_2_S will be hard, and most researchers exhibit discharge property lower than 80% of the theory ceiling. To overcome the above issue, it is essential to introduce conductive MOFs or hybridize them with other conductive materials (electroconductive polymers, porous carbon, etc.) to reduce internal resistance and promote fast electrons delivery.

**Figure 7:**
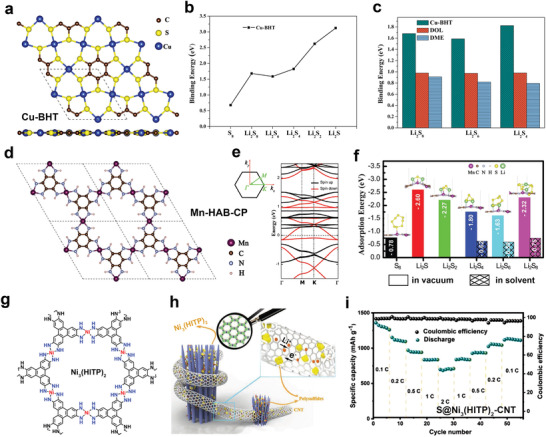
a) The structure and b) binding energy of conductive Cu‐BHT for LSBs. c) The comparison of the binding energy of Cu‐BHT, DOL, and DME. a–c) Reproduced with permission.^[^ ^144^ ^]^ Copyright 2018, American Chemical Society. d) The geometry structure, e) bond structure, and f) the adsorption energy of polysulfides on Mn‐HAB‐CP. d–f) Reproduced with permission.^[^ ^145^ ^]^ Copyright 2018, Wiley‐VCH GmbH. g) The structure of Ni_3_(HITP)_2_. h) Composites of conductive Ni_3_(HITP)_2_ with CNTs and i) their rate performances in LSBs. Reproduced with permission.^[^ ^146^ ^]^ Copyright 2019, Wiley‐VCH GmbH.

Recently, electrically conductive MOFs have drawn significant attention as promising materials for MSBs, mainly owing to the combination of high porous nature and electrical conductivity. Meanwhile, remarkable striving has been devoted to advance MOFs with improved conductivities, such as in‐plane π‐conjugation form, through‐space or through‐bond electron transport, and doping.^[^ ^140^, ^141^, ^142^, ^143^ ^]^ For example, Zhao and co‐workers take advantage of through‐bond charge transport to construct MOFs with high conductivity,^[^ ^144^ ^]^ thus resulting in limitless metal‐sulfur chains with the promising tendency in electron transport (**Figure** **7**a). This investigation also showed that the high conductive Cu‐BHT monolayer possessed high S utilization for LSBs (Figure 7b,c), mainly due to the synergic interaction upon Li—S_BHT_ and S_LiPS_—Cu bonds, as well as the high conductivity. Besides, the in‐plane π‐conjugation formalism, as one promising structure leading to electric conductivity, has been confirmed by the Wang group.^[^ ^145^ ^]^ Based on their calculation, the 2D hexaaminobenzene‐based coordination polymers (Mn‐HAB‐CP) showed high metallicity owing to the effect of d–p–π conjugation and the strong adsorption ability towards polysulfides because of the exposure of both Mn and N sites (Figure 7d–f). Recently, 2D MOF, Ni_3_(HITP)_2_, which shows high conductivity, has been applied as S host materials in Li–S batteries (Figure 7g).^[^ ^146^ ^]^ Herein, an improving electrochemical performance was studied via combining Ni_3_(HITP)_2_ with CNTs, in which the CNTs provided long‐range electronic and ionic pathways, while Ni_3_(HITP)_2_ endowed the cathode short‐range electronic channels along with exceptional polysulfide shuttling suppression (Figure 7h–i). Moreover, the iodine‐doped sulfurized polyacrylonitrile was designed as S hosts in Na–S and K–S batteries, where the iodine doping could significantly enhance the conductivity of MOFs by producing organic metal iodide.^[^ ^147^ ^]^

**Figure 8:**
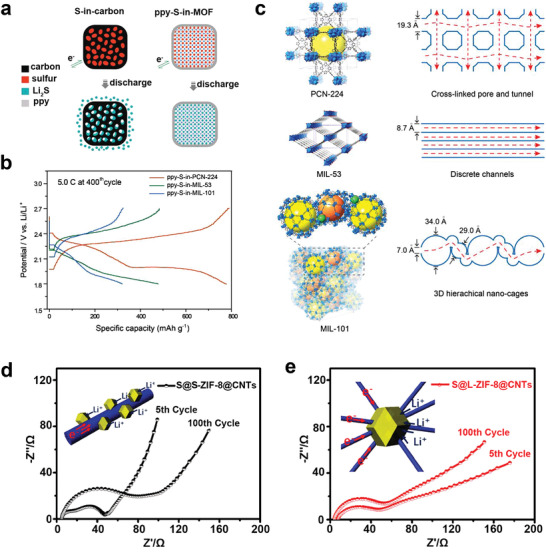
Schematic images of a) conductive MOF@PPy nanocomposites, b) MOF@PPy for LSBs, c) electron transfer in MOFs. a–c) Reproduced with permission.^[^ ^31^ ^]^ Copyright 2018, Wiley‐VCH GmbH. d) The ions and electrons transport with S@S‐ZIF‐8@CNTs and S@L‐ZIF‐8@CNTs in LSB. d,e) Reproduced with permission.^[^ ^32^ ^]^ Copyright 2018, Wiley‐VCH GmbH.

Additionally, MOFs have been hybridized with other conductive materials to prepare composite electrodes to settle the significant challenges of the inferior electroconductibility and structure instability during cycling, such as the conducting polymer, CNT, graphene, etc.^[^ ^148^, ^149^, ^150^, ^151^, ^152^ ^]^ Among all materials, conductive macromolecules (PANI, PPY, PEDOT, etc.) have been extensively explored owing to their high conductivity and strong chemical confinement on polysulfide intermediates.^[^ ^153^, ^154^, ^155^, ^156^, ^157^ ^]^ For example, the Deng group has integrated the merits of conductive polymer with MOFs to fabricate polypyrrole/MOF (PPY‐MOF) compartments (**Figure** **8**a,b).^[^ ^31^ ^]^ By further varying the pore geometries, MOFs with cross‐connected pores and channels showed the highest rate property due to their short mass diffusion distance and suitable pore size (Figure 8c). Recently, hollow MOF composites with PPY coating on ZIF‐67 hollow frameworks have been demonstrated to obviously increase the specific capacity and long‐term stability in LSBs.^[^ ^158^ ^]^ Meantime, they find that MOF composites with 60% S loading possess the highest electrochemical performance.

CNTs with high conductive and low‐dimensional nanoarchitectures can offer effective electron transport paths, resulting in abundant studies of MOF@CNT‐based electrodes. Recently, the Cao group presented a 3D MOF@CNT hybrid structure,^[^ ^32^ ^]^ where a freestanding 3D porous electrode was generated by grafting MOFs onto CNT sponges with particle size tailored to be 40 nm (S‐MOF@CNTs) or 500 nm (L‐MOF@CNTs). As a result, the L‐MOF@CNTs showed much‐increased cycling durability and rate performance than the S‐MOF@CNTs (Figure 8d–e), which mainly ascribed to the detachment and pulverization of S‐MOF@CNTs nanoparticles. Subsequently, the same group further evaluated the electrochemical performance of a sequence of CNTs@MOF with varying MOFs contents,^[^ ^159^ ^]^ where the moderately MOFs loading (≈70 wt%) showed the superior property. Because it ensured a thick electrode possessing high sulfur content and low polarization, yet still generating many small macropores to promote enough electrolyte infiltration.

## 5 Catalytic Conversion of Polysulfides by MOFs‐Derived Nanostructures

In a typical reaction in MSBs (M = Li, Na, K), sulfur (cyclic‐S_8_) reacts with metal ions to convert into long‐chain polysulfides (M_2_S*_n_*, 6 < *n* < 8) to shorter‐chain polysulfides (M_2_S*_n_*, 2 < *n* < 6) to finally M_2_S during cycling. Although the transformation of long‐chain polysulfides to shorter‐chain polysulfides is usually rapid, an increasing number of sulfurs in the cathode can weaken the transformation dynamics and extend the polysulfides’ residence time, thus leading to severe losses of active materials. Reaction kinetics enhancements can shorten the polysulfides’ detention time, thereby suppressing dissolution.^[^ ^13^ ^]^ Specifically, the slow transformation of polysulfides on the host materials results in saturated adsorption of polysulfides. Thus, the subsequent polysulfide confinement is restricted, and the dead sulfur amount rises.^[^ ^160^ ^]^ In contrast, the fast transformation of polysulfides on active materials results in the unsaturated state towards polysulfides, so the subsequent polysulfide confinement is feasible. Meanwhile, when M_2_S_2_/M_2_S is transformed into soluble polysulfides during the charging process, large additional driving is demanded. Owing to their ionic/electric insulative and insoluble feature in the aprotic electrolyte, the M_2_S_2_/M_2_S oxidation kinetics is sluggish and sulfur utilization is low.^[^ ^161^, ^162^ ^]^ Therefore, the enhancement of reaction kinetics to decrease the energy barrier of M_2_S_2_/M_2_S to polysulfides is anticipated to make the best utilization of sulfur. Until now, various active materials have been synthesized to accelerate the reaction kinetics.

### 5.1 Pristine MOF‐Derived Nanostructures for Catalytic Conversion

#### 5.1.1 Structural Designs

**Figure 9:**
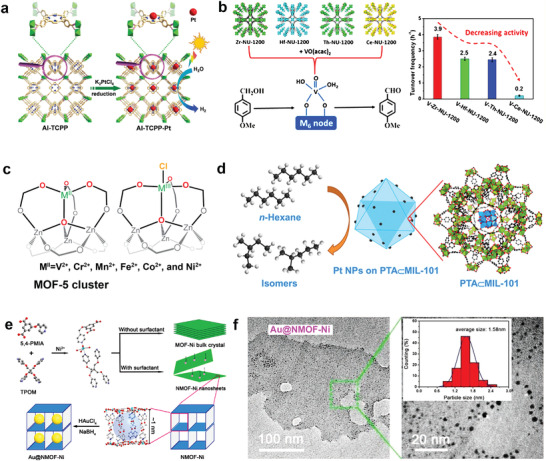
a) The synthesis of Al‐TCPP‐Pt. Reproduced with permission.^[^ ^168^ ^]^ Copyright 2018, Wiley‐VCH GmbH. b) Structure and activity of M‐NU‐1200. Reproduced with permission.^[^ ^169^ ^]^ Copyright 2019, American Chemical Society. c) MOF‐5 cluster. Reproduced with permission.^[^ ^52^ ^]^ Copyright 2013, American Chemical Society. d) Structure of PTA⊂MIL‐101 (PTA = phosphotungstic acid). Reproduced with permission.^[^ ^172^ ^]^ Copyright 2017, American Chemical Society. e) Scheme and f) TEM of Au@NMOF‐Ni. Reproduced with permission.^[^ ^174^ ^]^ Copyright 2018, Wiley‐VCH GmbH.

Generally, the rapid electrochemical reaction dynamics are induced via mild binding of polysulfides with cathode hosts, as well as effective electron transfer among them.^[^ ^163^ ^]^ MOF material provides an excellent opportunity to solve the critical problems of slow kinetics during cycling. Compared with traditional inorganic porous catalyst and nanometer catalyst, MOFs with evenly dispersed catalytic sites, large specific surface area, etc., offer better catalytic performances.^[^ ^164^, ^165^, ^166^, ^167^ ^]^ It is feasible to design MOFs with different hierarchies and rich polarity/catalytic sites. For example, the Jiang group has developed an aluminum‐based porphyrinic MOF (Al‐TCPP) to stabilize a single platinum site through strong chemical affinity with pyrrolic N atom,^[^ ^168^ ^]^ which provide highly effective charge transfer tunnels and thereby possess potential application in electrocatalysis (**Figure** **9**a). Recently, Farha and co‐workers investigated a sequence of isomorphic porous MOFs based upon transition, lanthanide, and actinide metals (such as Zr, Hf, Ce, Th, etc.), which were subsequently used as hosts for vanadium catalysts (Figure 9b).^[^ ^169^ ^]^ Notably, support effects were found when used these MOFs to catalyze alcohol oxidation, where the reactivities were linked to their electronegativity and oxidation state of the host metals, which was in accordance with the mechanism of polysulfides catalytic conversion.^[^ ^26^ ^]^ Similarly, Dincă group demonstrated that MZn_3_O(O_2_C—)_6_ cluster in MOF‐5 could be employed as supports for V^2+^ and Ti^3+^ through postsynthetic ion exchanges, where other MOF‐5 analogs with Cr^2+^, Cr^3+^, Mn^2+^, or Fe^2+^ at their metal sites could be attained via similar strategy (Figure 9c).^[^ ^52^ ^]^ Significantly, it was proved that electron transfer by the metal centers could be achieved in MOF‐5 analogs and portended novel redox reactivity at MOF secondary building units.

Typically, metal nanoparticles (NPs) are robust electrocatalysts and have also been broadly identified as effective catalysts in a great deal of electrochemical energy conversion technologies.^[^ ^34^, ^170^ ^]^ The Arava group has compared Pt, Au, and Ni nanoparticles’ catalytic activities in LSBs, where Pt NP‐coated Al foil showed significantly enhanced reaction kinetics.^[^ ^171^ ^]^ Therefore, designing and combining metal NPs with MOFs could provide unique catalytic sites. Meanwhile, the Yaghi group used MIL‐101 as support materials to encapsulate phosphotungstic acid in the framework's pores and depositing well‐defined platinum nanoparticles on the external surface (Figure 9d),^[^ ^172^ ^]^ leading to a highly active and selective bifunctional catalyst. Importantly, embedding Pt NPs inside MOF pores was achieved, which exhibited excellent catalytic activity due to uniform distribution and full utilization of Pt NPs.^[^ ^173^ ^]^ Additionally, Au NPs (≈1 nm) have also been incorporated inside the pores of nickel‐based MOF nanosheets (Figure 9e,f), which provide a sufficient catalytic surface and fast diffusion path.^[^ ^174^ ^]^ As a result, the introduction of catalytic metal sites or NPs in MOFs nanostructures may offer effective chemisorption and rapid electron transportation between polysulfides and the host framework.

#### 5.1.2 Applications in MSBs

**Figure 10:**
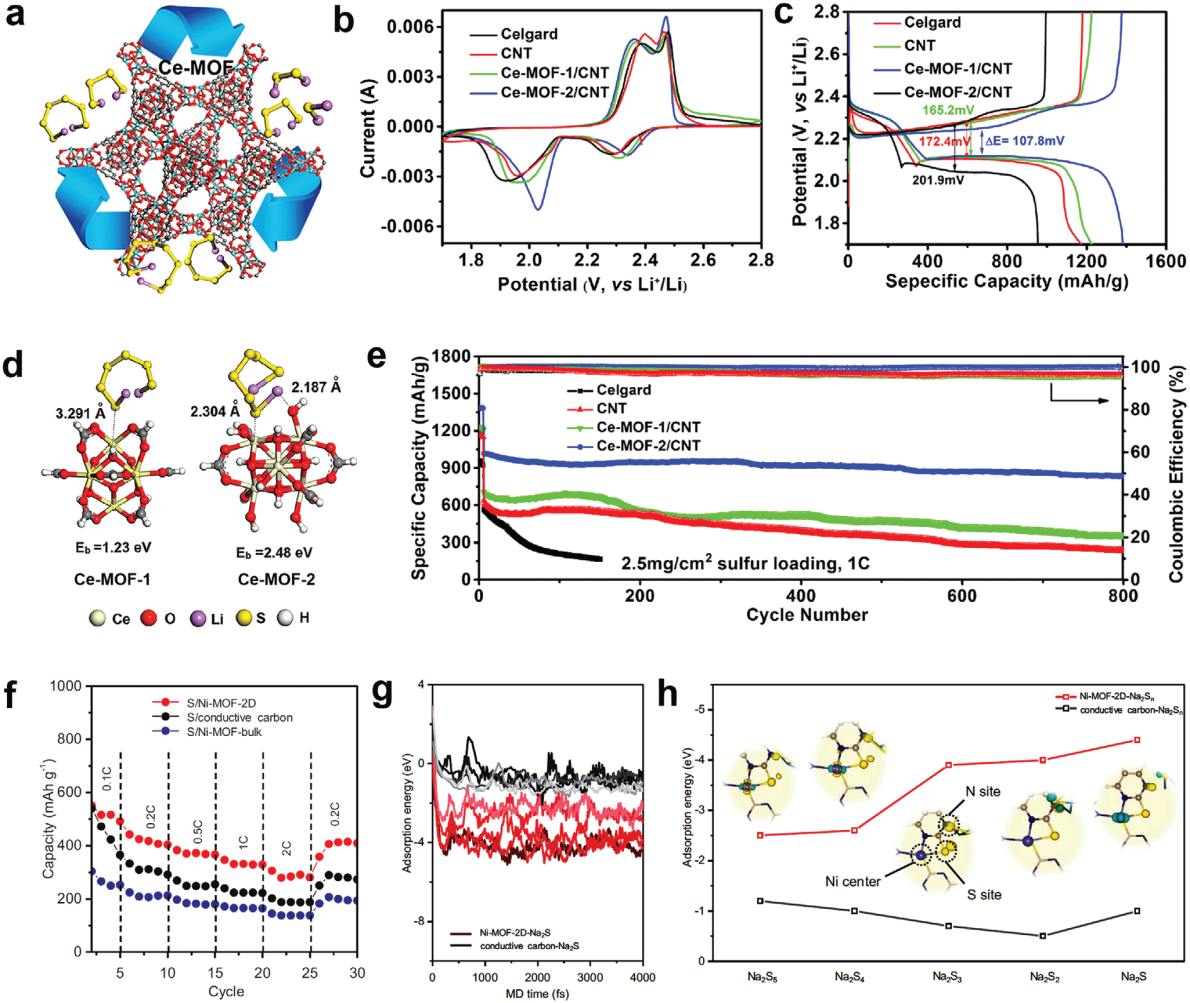
a) The scheme of Ce‐MOF catalyzed LSBs. b) The CV and c) charge–discharge performance of Ce‐MOF. d) Binding energy of Ce‐MOF with polysulfides. e) Ce‐MOF/CNTs catalyzing LSBs. a–e) Reproduced with permission.^[^ ^30^ ^]^ Copyright 2019, American Chemical Society. f) The rate performance of Ni‐MOF in NSBs. g) Adsorption energy versus ab initio molecular dynamics simulation time, and h) adsorption energies of charge difference analyses of Ni‐MOF in NSBs. f–h) Reproduced with permission.^[^ ^28^ ^]^ Copyright 2020, Wiley‐VCH GmbH.

It is regarded that the conversion of soluble Li_2_S_4_ to solid Li_2_S is the rate‐limiting step in an LSB, which results in three‐quarters of its theoretical capacity.^[^ ^175^ ^]^ However, on most occasions, the chemically bound polysulfides are blocked. Simultaneously, the external surface transformation cannot be directly achieved in the polar support, in which extra surface diffusion steps should be adopted for the whole reaction to run.^[^ ^176^, ^177^ ^]^ In contrast to the conventional inorganic porous catalysts or polar hosts, MOFs hold better catalytic property because of their evenly dispersed active sites and higher surface areas, which feature adequate binding ability between polysulfides and cathode host, as well as efficient charge transfer among them. For example, Cai and co‐workers prepared a Ce(IV)‐cluster‐contained MOF that was subsequently coupled with CNT to produce Ce‐MOFs/CNT hybrids to serve as separator coating in LSBs (**Figure** **10**a–c).^[^ ^30^ ^]^ Notably, the Ce‐MOFs that held high specific surface areas and coordination‐unsaturated Ce(IV)‐cluster sites could moderately interact with polysulfides and thereby promote their transformation (Figure 10d,e). Meanwhile, the generated Ce‐MOFs/CNT hybrids exhibited superior electrochemical properties due to the dual‐functional effects of catalyzing polysulfides conversion by restraining them from dissolving. More recently, the Qiao group proposed a new theory in polysulfides catalytic conversion based upon a 2D Ni(II)‐contained MOF.^[^ ^28^ ^]^ Electrochemical tests and systematic DFT computations revealed that the effective polysulfide confinement and rapid polysulfide conversion dynamics could be achieved via adjusting Na‐N/S interaction through dynamic electron state of Ni center (Figure 10g‐h). Remarkably, the electrochemical properties of the generating S cathodes were presented to be better than those of all S cathode hosts of NSBs (Figure 10f).

### 5.2 MOF‐Derived Catalytic Metal Compounds

#### 5.2.1 Structural and Material Designs

As mentioned above, adequate binding and fast charge transmission between polysulfides and cathode hosts can be the major factors to enhance the interfacial redox dynamics of MSBs. Particularly, transition metallic compounds, including transition metallic oxides, sulfides, nitrides, phosphides, etc., are promising materials owing to their fascinating physicochemical performances relying on varying metal cations and inorganic anions.^[^ ^178^, ^179^, ^180^, ^181^, ^182^, ^183^, ^184^, ^185^, ^186^, ^187^ ^]^ Most transition metallic compounds possess conductivity and polar surfaces applicable for catalytic reaction with sulfur species, therefore, occupying the most significant part in catalytic hosts for MSBs.

**Figure 11:**
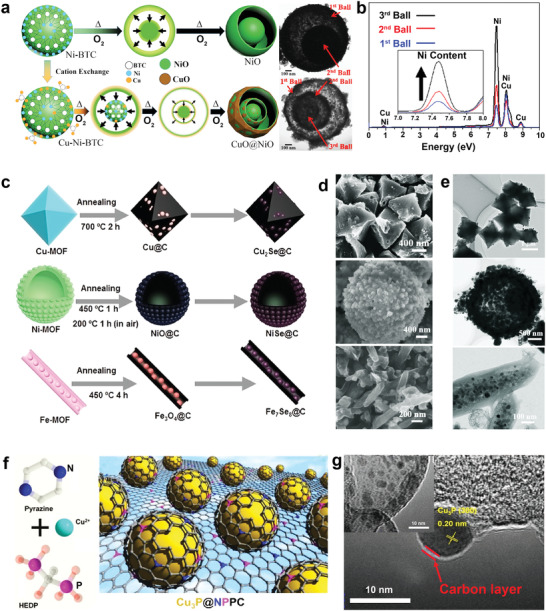
a) The SEM and b) EDS spectrum of the multishell hollow architecture of CuO@NiO. Reproduced with permission.^[^ ^188^ ^]^ Copyright 2015, American Chemical Society. c) The scheme images, SEM d), and TEM e) of Cu_2_Se@C porous octahedra NPs, NiSe@C hollow 0‐D microspheres, and peapod‐like 1‐D Fe_7_Se_8_@C nanorods derived from MOFs. c–e) Reproduced with permission.^[^ ^193^ ^]^ Copyright 2018, Wiley‐VCH GmbH. f) The structures and g) TEM of Cu_3_P@NPPC catalysts. f,g) Reproduced with permission.^[^ ^198^ ^]^ Copyright 2018, Wiley‐VCH GmbH.

Designing potential transition metal oxides with the desired morphology should be effective strategies to acquire enhanced electrocatalytic activity. For example, the Wang group successfully synthesized multilayer hollow CuO@NiO spheres by a heterogeneous post‐annealing process of CuNi bimetallic MOFs in the air.^[^ ^188^ ^]^ The CuO@NiO products consisted of three ball‐in‐ball shells, and interlinked CuO/NiO particles were integrated to generate this multilayer architecture (**Figure** **11**a,b). There still existed an elemental gradient (most Cu locating on the external surface, while most Ni inside the internal core) in the resulted products, consistent with the Li^+^ ions insertion sequence of these metal oxides (CuO, NiO), which thus could potentially offer accelerated polysulfides transformation and multiple catalyses in MSBs. Similarly, the Lou group demonstrated a new strategy for the efficient preparation of box‐in‐box nanocages with diverse layer components, i.e., Co_3_O_4_/NiCo_2_O_4_ double‐layered nanocages.^[^ ^189^ ^]^ Notably, the distinct shell structures, complex components, and the incorporation of Ni^2+^ inside their spinel architectures could lead to improved conductivity and new catalytic sites.

Introducing potential metal sulfides and selenides that possess sulfiphilic sites and higher electrical conductivities than metal oxides could realize superior catalytic activity.^[^ ^190^ ^]^ Recently, the Lou group reported a simple construction of composite nanoboxes with complex layer structure where the CoSe‐enriched internal shells were intimately limited inside the carbon‐enriched external shells (named as CoSe@carbon nanoboxes).^[^ ^191^ ^]^ Notably, the hollow voids inside the nanoboxes were in favor of tolerating large volumes expansion; meanwhile, the CoSe nanoparticles could be well protected by their external carbon shells, thereby offering persistent catalytic sites. Moreover, it has also been demonstrated that the confined small CoSe_2_ nanoparticles in macroporous carbon frameworks can provide wide channels for electrons/ions transformation and are in favor of exposing active sites for long‐term cycling.^[^ ^192^ ^]^ Besides, a sequence of excellent metal selenide‐based materials (Cu_2_Se, NiSe, Fe_7_Se_8_) with particular micro‐/nanostructures has been achieved using MOF precursors (Figure 11c–e),^[^ ^193^ ^]^ which possess small charge‐transfer resistances and accelerated dynamics originated from the pseudocapacitive effect.

It is proved that transition metal phosphides usually display metallic nature or even superconductivity, which favors the fast electrons transferring.^[^ ^194^, ^195^ ^]^ Meanwhile, metal phosphides can mildly bind with polysulfides, making it easy to form and break the S‐P bond in MSBs, thus enhancing the reaction kinetics.^[^ ^196^, ^197^ ^]^ Therefore, it is anticipated to develop potential metal phosphides to satisfy fast polysulfides conversion. The uniformly dispersed Cu_3_P nanoparticles incorporated inside an N, P‐codoped carbon layer (NPPC) has been reported (Figure 11f,g).^[^ ^198^ ^]^ Importantly, the cooperative effect of Cu_3_P sites with the polar heteroatom‐doping offers more possibilities to achieve fast charge migration and accelerated electrochemical kinetics on the Cu_3_P surface. Similarly, a porous 2D O‐incorporated CoPO ultrathin nanosheet has been synthesized to offer accelerated mass/electron transport to catalytic sites.^[^ ^199^ ^]^ Moreover, the unique structure combined with the O/P synergistically triggered the high‐oxidized Co to preserve strong electron‐donating P for promoting their electrocatalytic activities.

#### 5.2.2 Applications in MSBs

##### Metal Oxides

MOFs consisting of diverse metal ions and organic linkers can easily produce metal oxides during pyrolysis in the air. Until now, two mechanisms of metal oxides catalyzing polysulfide conversion have been raised. One is forming surface‐bound thiosulfate/polythionate mediators by metal oxides reacting with polysulfides.^[^ ^200^ ^]^ It means that metal oxides first oxidize the initially formed polysulfides to undissolving thiosulfate groups on metal oxides’ surfaces. Subsequently, the newly formed polysulfides were immobilized via S–S interactions with the thiosulfate groups, resulting in the construction of polythionate and then converting to undissolving products. For example, the conversion of polysulfides followed by thiosulfate generation has been found on the surfaces of CuO/VO_2_ with desired redox potential within the scope of 2.4–3.05 V.^[^ ^201^ ^]^ Generally, the redox potential for forming polysulfides was less than 2.4 V. Therefore, the slightly higher redox potential of metal oxide could promote polysulfide oxidation and subsequent thiosulfate generation. Nevertheless, unreactive sulfate species will be created on metal oxides when their redox potentials exceed 3.05 V because of excessive oxidation.

**Figure 12:**
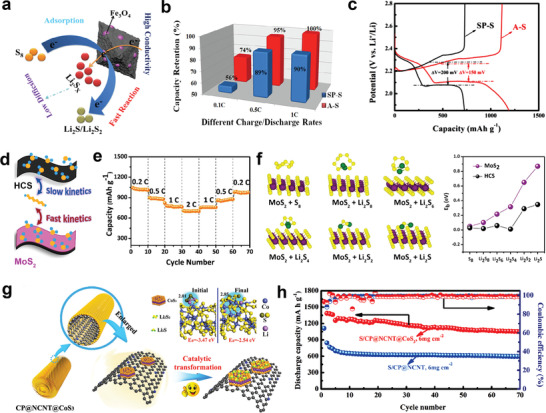
a) Charge and b) discharge process, stability, and c) charge–discharge performance of Fe_3_O_4_ electrode. a–c) Reproduced with permission.^[^ ^205^ ^]^ Copyright 2020, Elsevier B.V. d) MoS_2_ catalyzed LSBs. e) The rate performance and binding energy of MoS_2_ in LSBs. d–f) Reproduced with permission.^[^ ^217^ ^]^ Copyright 2018, The Royal Society of Chemistry. g) The scheme and DFT simulation of sulfur species transformation on CP@NCNT@CoS_3_ (NCNTs = nitrogen‐doped carbon nanotubes). h) The stability of CP@NCNT@CoS_3_ in LSBs. g‐h) Reproduced with permission.^[^ ^36^ ^]^ Copyright 2019, Wiley‐VCH GmbH.

Another mechanism of catalytic activities emphasizes the significance of synergistically promoting both the adsorption and diffusion of polysulfides on the mild polar surfaces of metal oxides.^[^ ^202^ ^]^ It is proved that robust absorption of polysulfides on polar surfaces is effective for property improvements, while surface diffusion from these nonconductive metal oxides into conductive carbon hosts is necessary to accept electrons.^[^ ^203^, ^204^ ^]^ For example, La_2_O_3_, MgO, and CeO_2_ presented superior capacities and stabilities than Al_2_O_3_ in LSBs.^[^ ^202^ ^]^ Despite the adsorption energy of polysulfides on Al_2_O_3_ being the highest, the sluggish surface diffusion decreased the redox dynamics and worsened the problems. Meanwhile, it has been revealed that the polar Fe_3_O_4_@C can efficiently control and regulate the redox reaction to restrain the shuttling via accelerating the conversion of polysulfides (**Figure** **12**a–c).^[^ ^205^ ^]^ They conclude that the Fe_3_O_4_@C can ensure rapid electron/ion migration and confine polysulfide molecules within the cathode via conductive frameworks and moderately adsorption capacity. Consequently, mildly bonding with polysulfides and subsequent easy surface diffusion on the non‐conductive metal oxides will result in superior sulfur hosts.

##### Metal Sulfides

Since transition metal sulfides have been employed as hydrodesulfurization catalysts to decrease the S content of refined oil products, plenty of metal sulfides are investigated to accelerate redox dynamics in MSBs. Most researchers summarized that metal sulfides possess sulfiphilic sites for robustly interacting with polysulfides and higher electroconductibilities than metal oxides because of their delocalized electronic microstructures.^[^ ^206^, ^207^, ^208^, ^209^, ^210^, ^211^, ^212^, ^213^ ^]^ When TiS_2_, ZrS_2_, and VS_2_ were employed as cathode materials, they were able to promote fast charge transfer and mild bonding with polysulfides.^[^ ^214^ ^]^ As a result, reduction and oxidation reactions of polysulfides were accelerated during cycling, thereby resulting in superior rate capabilities.

A significant discrepancy from metal oxides should be the 2D‐layered architectures in metal sulfides containing two atomic configurations (namely basic plane and edge sites). Generally, when 2D layered metal sulfides were applied as catalysts in usual industrial domains, the reactive activation primarily originated from active edge sites.^[^ ^215^, ^216^ ^]^ It has been proved that the sulfur in sulfides can mildly bind with lithium in polysulfides, thereby weakening reacting energy barriers and accelerating electrochemical redox kinetics (Figure 12d–f).^[^ ^217^ ^]^ The Cui group has proposed that the transformation of soluble polysulfides to insoluble productions can selectively take place along the edge sites of MoS_2_ owing to their stronger binding energies with Li_2_S than that of the basal plane.^[^ ^218^ ^]^ The redox dynamics improvement at edge sites has been further explored with WS_2_ and MoS_2_.^[^ ^219^ ^]^ They observed that coordination unsaturated atoms on the edge sites were in favor of charge transfer and interaction with sulfur species. From this perspective, the Lee group prepared a sulfur‐defective MoS_2_ and reduced graphene oxide composites (MoS_2_‐*x*/rGO) to form the accessible Mo atoms in the basic plane.^[^ ^220^ ^]^ Cyclic voltammetry result of MoS_2_‐*x*/rGO presented a relatively lower polarization and higher specific current, as well as more segregated oxidative/reductive peaks in contrast to that of MoS_2_/rGO, revealing the excellent catalytic activity of the defective metal sulfides.

Apart from 2D layered metal sulfides, another formalization of pyrite‐type metal sulfides, including CoS_2_, FeS_2_, and etc., was also investigated.^[^ ^35^, ^37^, ^41^, ^221^ ^]^ Amorphous CoS_3_ has been utilized as an efficient catalyst for the fast solid–solid transformation of Li_2_S_2_ to Li_2_S (Figure 12g,h).^[^ ^36^ ^]^ They found that the CoS_3_ possessed robust bonding with the sulfur species, hence rendering the immobilizing of S species on its external surface and ensures the invertible conversion among S species. In the meantime, the Li_2_S_2_ displays lower diffusion obstruction on the surface of CoS_3_, suggesting a facilitated transformation of Li_2_S_2_ to Li_2_S. As mentioned above, the crucial features for improving the redox dynamics are the fast charge transfer and suitable polysulfide binding capacity. Cui group have methodically explored a sequence of metal sulfides (CoS_2_, VS_2_, SnS_2_, FeS, TiS_2_, and Ni_2_S_3_) to expose the related theory for catalytically decomposing Li_2_S.^[^ ^46^ ^]^ The decomposition evolution was concluded involving a Li_2_S molecule cleaving to a single Li^+^ ion and a LiS anion, and the primary process can be the Li^+^ moving away. The calculation revealed that the order of the magnitude of the Li_2_S dissociation obstacle on metal sulfides was Ni_3_S_2_ > FeS > CoS_2_ > SnS_2_ > VS_2_ > TiS_2_, consistent well with the potential estimated experimentally. Meanwhile, the dissociation pathway demonstrated that the dissociation evolution related to the interaction between S in sulfides and the dissociative Li^+^, which could be the governing factor for inferior dissociation obstacle compared with traditional carbon host materials.

##### Metal Nitrides/Phosphides

**Figure 13:**
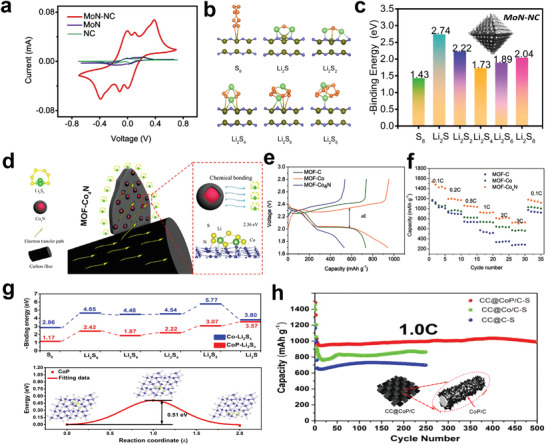
a) The symmetric battery of MoN. b) The interaction and c) adsorption energy between MoN and polysulfides in LSBs. a–c) Reproduced with permission.^[^ ^228^ ^]^ Copyright 2019, The Royal Society of Chemistry. d) The confinement of Co_4_N towards polysulfides in LSBs. e) The charge–discharge performance and f) rate performance of Co_4_N. d–f) Reproduced with permission.^[^ ^229^ ^]^ Copyright 2019, WILEY‐VCH GmbH. g) The adsorption and dissociation energy between CoP and polysulfides and h) its cycle life in LSBs. g,h) Reproduced with permission.^[^ ^39^ ^]^ Copyright 2019, Wiley‐VCH GmbH.

Lately, metallic compounds, such as nitrides and phosphides, have also been widely researched as electrocatalysts for MSBs because of their metallic nature and strong polysulfides confinement, which enable rapid surface reactions.^[^ ^222^, ^223^, ^224^, ^225^ ^]^ For example, it was found that transition metal nitrides, involving ScN, TiN, VN, CrN, and MnN, exhibited much stronger bonding ability towards polysulfides than conventional porous carbon materials and corresponding metal sulfides due to both the surfaces of transition metals and N atoms could participate in the interaction with polysulfides.^[^ ^226^, ^227^ ^]^ Besides, calculation revealed that inferior ion diffusion obstructions and activation obstructions of Li_2_S dissociation could be achieved on the surfaces of transition metal nitrides, which was significant to accomplish highly invertible capacity and fast reaction dynamics. In this respect, MoN was also revealed to possess moderate adsorption ability, and the CV for the symmetrical batteries showed sharp peaks and narrow separation in each redox pair,^[^ ^228^ ^]^ which suggested high activities for converting polysulfides. (**Figure** **13**a–c). Similarly, the Co_4_N nanoparticles have been embedded into a 2D N‐doped carbon structure, which provide strong chemical absorption ability and catalytic surfaces. In contrast, the 2D conductive N‐doped carbon framework guaranteed accelerated charge transfer, thus resulting in enhanced cycling stability and rate property (Figure 13d–f).^[^ ^229^ ^]^

Compared with the low conductivity of most transition metal oxides and sulfides, the transition metal phosphides show metallic features and even superconductivity, which are in favor of the charge transfer in MSBs.^[^ ^230^, ^231^ ^]^ More importantly, Qian and co‐workers concluded that the p orbits coming from the nonmetallic anions could regulate the interfacial redox kinetics via modulating the valence electron energy.^[^ ^232^ ^]^ They found that compared with Co_3_O_4_ and CoS_2_, the p band center in phosphating cobalt had an obvious upshift relative to Fermi level, thereby rendering a shrinking energy gap within the cobalt 3d and phosphorus 2p band centers. This further resulted in higher electron energy, facilitating the electron exchange to accelerate interfacial S_6_^2–^/S^2–^ redox dynamics. Liu and co‐workers recently systematically studied the electrochemical features of Li–S chemistry with Fe_3_O_4_ and FeP based on electrochemical tests and DFT computations.^[^ ^233^ ^]^ Impressively, tests revealed that the p band center of the FeP was markedly shifted to the Fermi level, which exhibited similar behavior to CoP, suggesting enhanced kinetics could be realized via achieving increasing electron energy in metal phosphides.

A shrunken energy gap within bonding and antibonding orbitals enables the P atom much more easily coupling with or separating from other atoms,^[^ ^229^ ^]^ making it easy to form and break the S—P bond in MSBs, resulting in enhanced kinetics. Liu and co‐workers designed and synthesized well‐defined carbon‐encapsulated CoP nanosheets arrays for LSBs, where the mild interaction between polysulfides and active materials should be key factors to accelerate kinetics.^[^ ^39^ ^]^ Notably, DFT was performed to disclose the essential interfacial regulation principle of cobalt and CoP compounds, in which the Co (111) has more negative binding energy than the CoP (211) surface, suggesting relatively easy polysulfides transformation for CoP (Figure 13g,h). Meantime, a lower diffusion energy barrier of Li_2_S on CoP was disclosed, which meant that Li_2_S could transform easily on the CoP surface. To deep recognize the chemical interactions between CoP and polysulfides, the Wang group proposed that CoP could strongly confine polysulfides due to their natural oxidation abilities (Co—O—P‐like formalization) that activated the surficial Co sites for confining polysulfides based on a robust Co—S bond.^[^ ^234^ ^]^ This surficial oxidation layer enables strong polysulfide binding and an internal core appropriate for transferring charge, resulting in highly stable cycling performance. Moreover, it is found that other metal phosphides and chalcogenides such as Ni_2_P, FeP, MoP, CoS, CoSe_2_, etc., all relied on the surface oxidation layer for chemically binding polysulfides, suggesting the surface oxidation‐activated polysulfides confining principle could be a universal phenomenon and could uncover the mechanism of numerous transition metal phosphides/chalcogenides in hosting S cathodes.

### 5.3 Single‐Atom Catalysts

#### 5.3.1 Structural Designs

**Figure 14:**
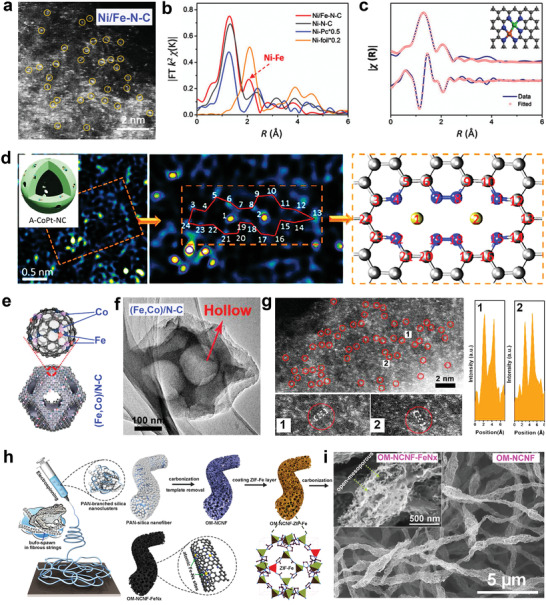
a) The HAADF‐STEM images, b) the Fourier transformation of the EXAFS spectra, and c) the first two shell (Ni–N, Ni–Fe) fitting of Ni/Fe–N–C. a–c) The structure of Ni/Fe‐N‐C sites. Reproduced with permission.^[^ ^241^ ^]^ Copyright 2019, WILEY‐VCH GmbH. d) The structure and bright‐field STEM image of CoPt–N–C. Reproduced with permission.^[^ ^242^ ^]^ Copyright 2018, American Chemical Society. e) The scheme image, f) HRTEM image, and g) HAADF‐STEM images of Co/Fe–N–C. e–g) Reproduced with permission.^[^ ^247^ ^]^ Copyright 2017, American Chemical Society. h) The scheme and i) SEM of OM‐NCNF‐FeN*_x_* (atomic Fe–N*_x_* coupled open‐mesoporous N‐doped‐carbon nanofibers) with a hierarchical porous structure. h,i) Reproduced with permission.^[^ ^248^ ^]^ Copyright 2018, WILEY‐VCH GmbH.

Single‐atom catalysts (SACs), composed of well‐dispersed single atoms hosted on diverse matrixes, optimize the atomic effectiveness and enable each atom to contribute, which provide a promising approach to regulate the activity and selectivity during a catalytic process.^[^ ^235^, ^236^, ^237^, ^238^, ^239^, ^240^ ^]^ Therefore, to efficiently meet the requirements of fast conversion in MSB, potential single‐atom sites, especially dual single‐atom sites with desired structures, are required. For example, the Zhao group demonstrated a biatomic metal‐nitrogen site (Ni/Fe–N–C), through an ionic exchange process with the pyrolysis of Zn/Ni/Fe‐based ZIF (**Figure** **14**a–c).^[^ ^241^ ^]^ Besides, Yao and co‐workers proposed a novel kind of bi‐atomic Co–Pt carbon/N‐based catalyst, which directly employed the exposed defects on the shells of carbon capsules to generate Co–Pt–N–C coordination configurations as catalytic sites.^[^ ^242^ ^]^ The bi‐atomic metals (Co/Pt) were captured into vacancy‐type defects to generate integrality of Co–Pt–N–C coordination configurations (Figure 14d). Meanwhile, DFT results revealed that atom‐dispersed Pt–Me (Me = Co/Pt) combining at a carbon defect could markedly clip the electronic configuration of metal atoms and modify the electron distribution in the coordination configurations, leading to improved and synergetic activity.

To achieve fast electrons/ions transferring to active sites, charming architectures (hollow, hierarchical porous structures, etc.) are promising to incorporate with SACs.^[^ ^243^, ^244^, ^245^, ^246^ ^]^ The porphyrin‐like Fe–Co dual sites inside hollow nitrogen‐doped porous carbon material have been employed for electrocatalytic reactions (Figure 14e–g).^[^ ^247^ ^]^ Moreover, our group has utilized open‐mesoporous carbon nanofibers to couple with homogeneously Fe–N*_x_* sites to increase the redox dynamics of air cathodes (Figure 14h,i).^[^ ^248^ ^]^ The excellent electrocatalytic activity of this unique architecture could be ascribed to the following factors: i) the uniformly coordinated atomic Fe–N*_x_* configurations ensured their excellent reactivity and stability; ii) the high mesopores, interconnected architectures, and large surface areas not only fully exposed Fe–N*_x_* active sites but also accelerated the mass/electron transport performance; iii) the 3D hierarchical porous channels and frameworks in cathode observably improved the air diffusion pathways. As a result, it is believed that these fascinating SACs, especially dual or multiple single‐atom sites with desired porous structures, can provide massive possibilities for future higher performance and long‐life MSBs.

#### 5.3.2 Applications in MSBs

**Figure 15:**
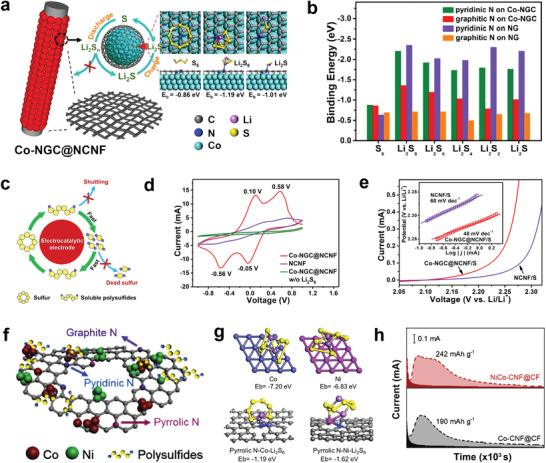
a) The DFT configuration confinement and b) binding energy of Co‐NGC@NCNF (Co‐embedded N‐doped graphitic carbon decorated on N‐doped carbon nanofibers). c) The catalytic process of Co‐NGC@NCNF in an LSB. d) The symmetric battery and e) Tafel slope of Co‐NGC@NCNF in LSBs. a‐e) Reproduced with permission.^[^ ^34^ ^]^ Copyright 2019, Elsevier B.V. f) The geometric construction, binding energy g), and Li_2_S deposition capacity h) of NiCo‐CNF@CF (CNF = carbon nanofiber, CF = carbon fiber). f–h) Reproduced with permission.^[^ ^259^ ^]^ Copyright 2019, Elsevier Ltd.

SACs possess single metal sites or atomic centers usually hold optimized atom utilization, unsaturated coordination centers, and distinct electronic configurations.^[^ ^249^, ^250^, ^251^, ^252^, ^253^ ^]^ Therefore, they have been intensively employed as electrocatalysts for energy conversion. Meanwhile, the remarkable electron configurations of SACs with distributed energy levels and concentrated open metal sites can effectively accelerate the dynamics conversion of polysulfides in MSBs.^[^ ^254^, ^255^, ^256^, ^257^, ^258^ ^]^ For example, a dual functional M–N–C electrocatalytic cathode comprised of Co‐inserted N‐doped graphitic carbon has been proposed for the electrocatalytic conversion of polysulfide (**Figure** **15**a,b).^[^ ^34^ ^]^ Notably, the DFT calculations revealed that the graphitic nitrogen interacted with cobalt sites in the carbon array exhibited moderate reactivity and could provide bifunctional active centers for stable yet facile adsorption/decomposition of polysulfides, which resulted in significantly decreased energy and dynamics barriers for fast polysulfide conversion (Figure 15c–e). Moreover, the dual active sites of Ni/Co–N–C coordination for effective sulfur anchor and conversion were investigated via a simple chemical vapor deposition strategy based on the Ni‐etched ZIF‐67 (Figure 15f).^[^ ^259^ ^]^ Importantly, the result interlayer materials possessed significant N‐doped (Co, Ni)–N–C heterostructures (Figure 15g‐h), which served as multiple active sites to ensure fast charge transfer and robust sulfur species adsorption/dissociation effect, resulting in reduced polysulfides shuttle effect and accelerated conversion kinetics.

**Figure 16:**
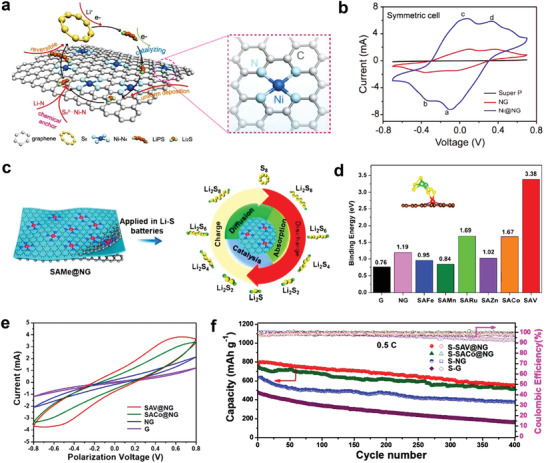
a) The catalysis and symmetric battery b) of Ni@NG in an LSB. a‐b) Reproduced with permission.^[^ ^262^ ^]^ Copyright 2020, WILEY‐VCH GmbH. c) The catalysis diagram and adsorption energy d) of S‐SAV@NG in an LSB. e) The symmetric battery. f) The cycle stability of SAV@NG. c–f) Reproduced with permission.^[^ ^44^ ^]^ Copyright 2020, American Chemical Society.

As mentioned above, the transition metal‐based SACs usually bond with nitrogen species to form an M–N–C configuration.^[^ ^260^, ^261^ ^]^ The binding energies and Gibbs free energy between polysulfides and metal‐N_4_/graphenes have been systematically calculated via the DFT method.^[^ ^45^ ^]^ Results revealed that the metal‐N_4_ structures exhibited moderate binding strength with soluble Li_2_S_n_ species coming from interactions of metal…S and N…Li bonds. Moreover, Niu and co‐workers successfully embedded single Ni atoms in N‐doped graphene, which could not only anchor the polysulfides firmly but also promote their conversion dynamics during cycling (**Figure** **16**a,b).^[^ ^262^ ^]^ It is found that the high‐valence Ni species in the Ni–N_4_ configuration could reversibly catalyze the conversion of the polysulfides relying on the S*_x_* ^2–^…Ni–N binding, leading to the inferior decomposition energy of Li_2_S, thereby enhancing the kinetic conversion of polysulfides. More recently, a synthetical investigation of 10 materials (graphene, NG, SAFe@NG, SAMn@NG, SARu@NG, SAZn@NG, SACo@NG, SAV@NG, SACu@NG, and SAAg@NG) has been proposed by Cui group to study the potential catalytic conversion mechanism towards polysulfides (Figure 16c,d).^[^ ^44^ ^]^ Importantly, the single V active sites showed improvement in both the adsorption and dissociation of sulfur species during the charge‐discharge processes (Figure 16e,f). The dramatically enhanced electrochemical property could be ascribed to the advantages of single atom induced electrode structure construction, including i) the single V sites could efficiently trap the soluble polysulfides and offer more easily decomposition of Li_2_S; ii) the graphene hosts promoted an accelerated charge transfer to catalytic sites and tolerated a relatively large volume variation during cycling; (iii) the facile effective catalytic conversion of Li_2_S to polysulfides accelerated the redox dynamics and prevented the generation of dead sulfur, thereby achieving high coulombic efficiency, fast kinetics, and long‐term stability.

### 5.4 Catalytic Heterostructures

#### 5.4.1 Structural Designs

**Figure 17:**
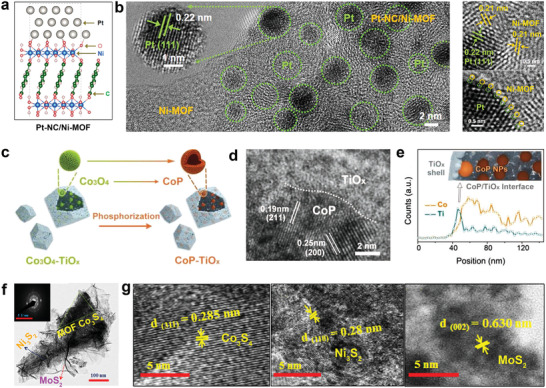
a) The schematic diagram and b) The heterostructure of Pt–NC/Ni–MOF. a,b) Reproduced with permission.^[^ ^265^ ^]^ Copyright 2019, Elsevier Inc. c) The schematic, d) TEM heterostructure, and e) the corresponding EDS of TiO*_x_*/CoP. c–e) Reproduced with permission.^[^ ^269^ ^]^ Copyright 2020, WILEY‐VCH GmbH. f) The TEM of Co_3_S_4_/Ni_3_S_2_/MoS_2_. g) The lattice fringe of Co_3_S_4_, Ni_3_S_2_, and MoS_2_. f,g) Reproduced with permission.^[^ ^270^ ^]^ Copyright 2020, Elsevier Ltd.

Heterostructured S cathodes built through unique interface design of diverse inorganic functional materials have drawn much interest in MSBs. Designing potential heterostructured sulfur hosts with an integrated polysulfide‐binding mechanism could realize promoted chemical interactions with polysulfides and accelerated interfacial polysulfide conversion dynamics.^[^ ^175^, ^263^ ^]^ Pt is usually considered a common choice with superior catalytic property towards many chemical reactions, rendering it dramatic for developing MOF/Pt multifunctional heterostructures. Zhu and co‐workers realized the in situ deposition of platinum nanoparticles on 2D MOF nanosheets through a non‐surfactant strategy.^[^ ^264^ ^]^ Remarkably, the existence of abundantly accessible O atoms within the defect‐rich surfaces of the ultrathin nanosheets enabled strong interfacial interactions through Pt‐O bonds. Meantime, the robust interfacial interactions in MOF@Pt heterostructures induced the compressive deformation of shortened Pt–Pt bonds, resulting in optimized d‐band centers and improved activities. Moreover, this type of strong interface interaction coming from the Pt–O bond was also demonstrated by the Qiao group.^[^ ^265^ ^]^ They proposed an interface‐bond‐induced intermediate regulation for promoting the HER and OER via combining 2D Ni‐based MOFs with platinum nanoparticles into heterostructures (**Figure** **17**a,b). Interestingly, experimental tests confirmed that the generation of a Ni–O–Pt bond electronically adjusted the heterostructures to tune the charge density of Pt and the high‐energy Ni 3d band, leading to optimized adsorption capacity for H* and OH*. Therefore, from the point of MSBs, the strong interface interaction of Pt–O bond in heterostructure may potentially realize fast polysulfides redox kinetics.

Additionally, the cooperative effect on the interphases between diverse metal compounds can modify the electronic structures of catalytic sites for adsorbing and activating the reactant molecules and optimize the configurations and reaction pathways of the generated intermediates, thus resulting in significantly improved reaction dynamics and rates.^[^ ^266^, ^267^, ^268^ ^]^ In this light, Zou and co‐workers presented a unique hollow structure that enabled uniformly distributed CoP nanoparticles on TiO*_x_* to form hybrid CoP/TiO*_x_* heterostructure (Figure 17c–e).^[^ ^269^ ^]^ Significantly, the heterostructured CoP–TiO*_x_* hybrid material could achieve the electron reapportionment on the CoP/TiO_x_ interfaces and optimize the electric density of CoP, which eventually results in accelerated OER dynamics. Meanwhile, the robust TiO*_x_* substrates in CoP–TiO*_x_* could immobilize the CoP NPs and avoid their aggregation, leading to highly accessible active sites. Besides, a triple‐phase heterostructure composing of metal compounds has been proposed.^[^ ^270^ ^]^ Remarkably, the tricomponent (MoS_2_, Ni_3_S_2_, and Co_3_S_4_) heterostructures could form effective interface architectures between the three components (Figure 17f,g), leading to promoted fast charge transfer of interphases to active catalytic sites. As a result, these advanced catalytic heterostructures constructed by noble metal nanoparticles or metal compounds can provide synergistic catalytic interfaces and superior electronic architectures of open active sites for ameliorative confinement and redox kinetics toward polysulfides.

#### 5.4.2 Applications in MSBs

**Figure 18:**
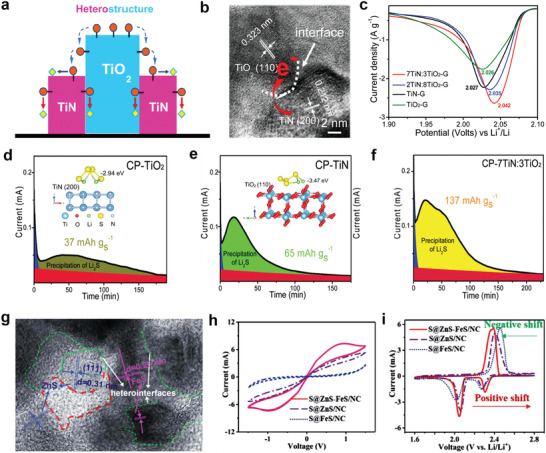
a) The schematic diagram and heterostructure b) of TiN/TiO_2_ in LSBs. c) CV performance of TiN/TiO_2_. d) The potentiostat performance of CP‐TiO_2_ (CP: carbon fiber paper), e) CP‐TiN, f) CP‐7TiN:3TiO_2_. a–f) Reproduced with permission.^[^ ^271^ ^]^ Copyright 2017, The Royal Society of Chemistry. g) The heterostructure of ZnS/FeS in LSBs. h) The symmetric battery and i) CV performance of ZnS/FeS. g–i) Reproduced with permission.^[^ ^278^ ^]^ Copyright 2020, The Royal Society of Chemistry.

Currently, metallic oxides possess powerful bond energies with polysulfides because of their conspicuous polarities, while surface diffusion is generally sluggish, and the charge transfer should be inferior. Meantime, metallic nitrides possess excellent conductivities, while their moderate absorption capacity toward polysulfides restrain the binding with soluble intermediates. In this regard, the desired heterostructure of TiO_2_/TiN was proposed by the Yang group. (**Figure** **18**a,b).^[^ ^271^ ^]^ This TiO_2_–TiN heterostructured sulfur cathode could accelerate the polysulfide conversion via three procedures: i) the soluble polysulfides were powerfully adsorbed on the TiO_2_ surfaces. Subsequently, the adsorbed polysulfides quickly diffused to the TiN. Moreover, at last, polysulfides were easily converted to insoluble species by fast charge transfer. As a result, the TiO_2_–TiN‐based cathodes exhibited long‐term cycle lives, excellent rate properties, and inferior electron transfer obstructions compared with TiO_2_ or TiN based cathodes (Figure 18c–f). Besides, other heterostructure‐based active materials, including MoN–VN,^[^ ^272^ ^]^ TiO_2_–MXene,^[^ ^273^ ^]^ VO_2_–VN,^[^ ^274^ ^]^ VO_2_–V_3_S_4_,^[^ ^275^ ^]^ TiN–sulfur doped oxide layer,^[^ ^225^ ^]^ TiC–graphene,^[^ ^276^ ^]^ and MoC–MoO*_x_*,^[^ ^277^ ^]^ also exhibited a synergistic effect of strong polysulfides capturing at active sites and effective electron transfer through conductive nodes. On the other hand, metal sulfides have been extensively employed as cathode materials in MSBs due to their facile synthesis process and strong chemical adsorption abilities towards polysulfide species. Zhang and co‐workers proposed ZnS–FeS heterostructures incorporated into N‐doped carbon with various inherent energy bandgaps involving 0.8 eV for FeS, while 4.9 eV for ZnS (Figure 18g).^[^ ^278^ ^]^ Therefore, a powerful E‐field in the heterointerface was generated due to the prominent energy bandgaps between the two components, which could efficiently facilitate polysulfide conversion. Moreover, the dynamics analyses and DFT computation also verified that the heterostructure‐rich cathodes could promote charge transfer, enhance the polysulfides confinement, and accelerate the surface redox dynamics, thus resulting in an outstanding rate capacity and favorable cycling stability (Figure 18h,i).

In order to achieve superior battery performance and long‐range stability of MSBs in practical applications, several key factors of the electrode materials are required: i) strong physical and chemical polysulfide confinement in cathode; ii) fast electron and ion transfer during charge and discharge process; iii) accelerated redox kinetics of lithium polysulfides; iv) high energy density are also required for scale‐up commercialization,^[^ ^64^ ^]^ MOFs with uniformly distributed polar sites and large specific area can achieve high areal sulfur loading while ensuring long‐term stability for MSBs.

3D free‐standing and highly conductive N, P co‐doped hydrogel‐derived carbon networks have been constructed to enhance the homogeneous dispersion of MOF nanodomains.^[^ ^18^ ^]^ This MOF‐based composite exhibits enhanced sulfur loading and capacity retention, where the areal sulfur loading amount can achieve as high as 18.8 mg cm^–2^ with a capacity retention of 82% after 300 cycles. The outstanding performance can be attributed to the physically trapping polysulfides of the hierarchically meso/microporous nanostructures and the enhanced charge transfer efficiency provided by MOF nanodomains. Moreover, Ce(IV)‐cluster nodes contained MOFs have been synthesized recently, which are then combined with CNT to form Ce‐MOFs/CNT composites to serve as the separator coating in LSBs.^[^ ^30^ ^]^ It is found that the Ce‐MOF‐2, which possessed a large specific surface area and coordination‐unsaturated Ce(IV)‐cluster nodes, can quickly adsorb the polysulfides and effectively catalyze their conversion. By combining the high conductivity of CNT, the synthesized Ce‐MOF‐2/CNT composite demonstrated excellent electrochemical and cycling performance with a high initial capacity of 993.5 mAh g^–1^ at ≈6 mg cm^–2^ sulfur loading with a capacity retention of nearly 100% upon 200 cycles was achieved at a 0.1 C discharging/charging rate.

Overall, MOF‐derived metal‐based nanostructures can realize both physicochemical adsorptions toward polysulfides and accelerate the redox kinetics owing to their accessible metal catalytic centers. The poor conductivity of pure MOFs inhibits their catalytic conversion performance even though they have exhibited good binding ability with polysulfides. Similarly, the MOFs‐derived metal oxides, nitrides, sulfides, selenides, and phosphides, are inherently polar materials with excellent catalytic active and strong binding strength with S species, which, however, most of them also have insufficient electrical conductivities and need additional conductive agents in MSBs. The MOF‐derived SACs with single metal catalytic sites usually hold optimized conductivity, atom utilization, unsaturated coordination centers, and distinct electronic configurations, which can effectively accelerate the catalytic conversion of polysulfides in MSBs. However, the design and scalable production of SACs for MSBs is difficult and expensive. Therefore, to balance MOF‐derived nanostructures’ performance and cost for MSBs, it is necessary to reasonably design the catalytic centers with cheap but superior conductive substrates. Moreover, enhancing the concentration of SACs as much as possible should be desired for achieving more efficient polysulfide catalysts.

## 6 Summaries and Future Perspectives

In summary, this review offers a conclusion of the recent advances of MOF‐derived nanostructures as multifaceted electrodes in MSBs. The recent progress is presented in designing MOF‐derived electrodes, including fabricating strategies, composition management, topography control, and electrochemical performance assessment. Particularly, we have systematically discussed the inherent charge transfer, intrinsic polysulfide immobilization, and catalytic conversion on designing and engineering of MOF nanostructures for efficient MSBs.

**Scheme 3:**
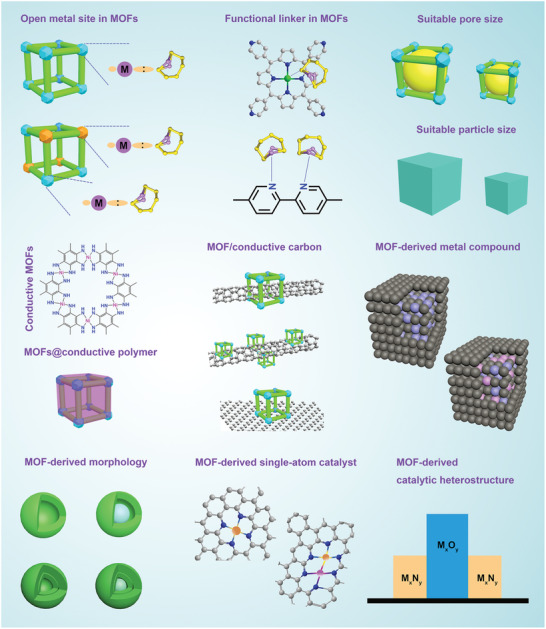
Schematic illustrations of diverse components and catalytic centers of MOF‐derived nanostructures for MSBs.

An ideal sulfur cathode should possess several essential features, including high specific surface area and porosity, easily accessible active sites, decent electron transfer, and mass transport capabilities, as well as durable stability. As a kind of new appearing porous materials, MOFs offer considerable advantages for designing diverse advanced electrode materials based on promising morphologies, structures, components, and performance. As for their intrinsic features, various synthetic methods have been proposed to acquire highly effective sulfur cathodes for MSBs, which can be commonly concluded as follows and also summarized in **Scheme** **3**.

First, benefiting from the high specific surface area, adjustable pore architecture, and various metal nodes/organic ligands, MOFs provide significant advantages to develop sulfur cathodes with improved capacity and long‐term lives for MSBs. The high specific surface areas can result in high S loading while their pores’ apertures optimize the confinement towards soluble polysulfides. Moreover, MOFs with polar functional groups and evenly dispersed catalytic sites, large specific surface area, etc., offer better catalytic performance towards polysulfides due to mild binding between polysulfides and cathode hosts, as well as effective electron transfer among them. Excellent electroconductibility can be another vital factor for improving the electron transfer number and electrochemical property. Stimulated by the high property of the conductive 2D MOF, Ni_3_(HITP)_2_, an increasing number of approaches, such as redox matching, in‐plane π‐conjugation, donor‐acceptor interactions, through‐space charge transport, and mixed‐valency formalization as well as developing 2D MOFs straight on conductive substrates, have been applied to enhance the electroconductibility, thereby resulting in higher capacity.

Second, MOF hybrids, combined with organic or inorganic multifunctional materials, have been investigated and effectively employed as S hosts in MSBs. These multifunctional composites generally own superior electroconductivity or catalytic activity and stability, including common carbon materials (such as heteroatom‐doped/undoped CNTs, graphene, and their hybrids), metallic nanoparticles, and conductive polymers. All components in MOF hybrids have unique individual functions. Hence, MOF composites can usually avoid the single counterparts’ shortcomings and induce cooperative effects for promoted polysulfides confinement and conversion. Furthermore, the MOF composites’ channels can serve as doors to efficiently control polysulfides and electrolytes, offering an excellent former for designing S hosts with selectable functions.

Third, inheriting the extraordinary characteristics of pristine MOFs in large part, the MOF derivatives possess particular opportunities compared with the conventional porous materials, involving the facile and effective synthetic process, high specific surface areas and porosity derived from pristine MOFs, easy to doping of uniformly distributed heteroatoms, and regulatable size/morphology/composition by pre‐designing MOFs/MOF composites. Hence, the extension of MOF/MOF composites creates a promising pathway to synthesize different highly effective S cathodes for MSBs. Besides, the organic linkers can be carbonized to form strongly conductive porous carbon substrates with uniformly doped heteroatoms, which guarantee the improved physical and chemical adsorption toward polysulfides and ensure the accelerated electron/mass transport and the accessibility of active sites. Moreover, the metal sites in MOFs array can be transformed into diverse inorganic materials by straight pyrolysis or posttreatment, including single atoms, metal nanoparticles/alloys, and metal compounds. Furthermore, in contrast to porous carbon materials, these multifunctional inorganic materials can be uniformly loaded in the resulting porous carbon substrates, thus increasing the concentration of active sites. Additionally, the binding strength with intermediates, break forms of S–S bonds can be well optimized, leading to enhanced polysulfide confinement and conversion.

To uncover the most promising MOF‐derived structures in designing advanced MSBs, Scheme 3 has presented the most representative nanostructures and catalytic centers of MOF‐derived materials. As for pristine MOFs, the most promising MOFs in MSBs should exhibit hierarchical porous structures and integrate with conductive substrates to guarantee large S loading, fast charge transfer, and suitable electron/mass transfer path; meanwhile, they should also display with multiple open metal sites and organic linkers with polar N/S/Se/P atoms to contribute superior chemical affinity/catalytic conversion activity towards polysulfides. After pyrolysis, MOF‐derived hierarchical porous carbon nanostructures with catalytic centers that integrated with single atoms and metal compounds (nitride, selenide, sulfide, and phosphide) may exhibit the most optimized battery performances. Recently, a strategy by embedding polar ZnS nanoparticles and Co–N–C SAC double‐end binding sites have been designed to enable a high‐energy and long‐cycling LSB.^[^ ^279^ ^]^ The synergetic design of catalytic centers can simultaneously promote the fast charge transfer and tune the chemical affinity/catalytic conversion property of polysulfides, thereby accelerating reaction kinetics in MSBs. Notably, the remarkable electron configurations of SACs with the elaborated design of atom centers, distributed energy levels, and concentrated open metal sites will become one of the mainstreams in exploring future high‐performance MSBs with promoted dynamics on polysulfides’ catalytic conversion.

Overall, though unbelievable progress has been accomplished, the advance of MOF‐derived nanostructures as multifaceted electrodes in MSBs is maybe still in the preliminary stage, and there still exist numerous issues for in‐depth exploration of more effective MOF‐derived nanostructures for electrodes in MSBs. Herein, some of the challenges are listed as follows.

1) Restricted usability of pristine MOFs for unique applications, involving strongly conductive and robust MOFs as electrode materials, and MOFs/MOF composites‐based metal‐free porous carbon derivatives.
2) Large‐scale preparation of MOFs. The high cost of organic linkers, severe reaction conditions during some synthesis, and a certain number of synthetic approaches harshly hinder the development progress from fundamental study to practical applications.
3) Theoretical researches are necessary for the unique synergistic effects in the improved catalytic activity of MOF hybrids and the derived inorganic functional materials.
4) Despite the fact that many recent studies have demonstrated that pristine MOFs can be straightly applied for polysulfide electrocatalysts, the surfaces of MOFs could suffer an irreversible phase transition during the charge‐discharge process. Hence, the actual catalytic sites should be verified via more advanced testing.
5) Trouble in synthesizing metal‐free carbon substances from MOFs. Remnants of metal species usually keep up in MOF derivatives even after an acidic etch.
6) Accurate regulation over the catalytic sites, especially in MOF‐derived materials. These derivatives generally include multiple phases because of the complicated evolution during carbonization or posttreatment processes.
7) Precise adjustment and management of pore architectures in MOF‐derived nanostructures. The carbonization often needs high temperature, which generates a hindrance in the reasonably controlled synthesis of derivatives with promising porous features.
8) Absence of comprehending of the conversion process from MOFs to the resulting derivatives.
9) The catalytic activity in MOF‐derived nanostructures needs to be further enhanced, both in polysulfide reduction reaction (pSRR) and polysulfide oxidation reaction (pSOR), which is highly eager for the actual commercialization of MSBs.

To conquer the above issues, some solutions and possible future tendencies could be listed below.

i) The as‐synthesized MOFs with robust metal–ligand coordination interaction, catalytic activity, in‐plane π‐conjugation effect, and through‐space or through‐bond electron transport are desired. The coordination interactions between metal nodes and organic linkers mainly predict MOF stabilities, while other factors determine the conductivity.^[^ ^114^, ^121^, ^140^, ^141^, ^142^ ^]^ Therefore, some MOFs species, including metalloporphyrin‐based MOFs, phthalocyanine‐contained in‐plane π‐conjugation MOFs, and the extensive UiO family, may exhibit promising trends as new electrodes for MSBs.
ii) Covalent organic frameworks (COFs), possessing intact organic networks, offer significant advantages to simply construct porous metal‐free carbon substances based on the accurate adjustment of heteroatom doping.^[^ ^280^, ^281^ ^]^
iii) More simple strategies for the preparation of MOF‐based substances need to be proposed. For instance, the postsynthetic modification of metal sites and/or ligands in MOFs, including the metal ions exchange and ligands incorporation followed by defect generation, can increase MOFs family members.^[^ ^282^, ^283^ ^]^ Designing abundant MOF hybrids (such as MOFs with polyoxometallates, metal‐based NPs, and conductive materials), while post‐processing of derivatives, should be the other two widespread strategies.^[^ ^284^, ^285^, ^286^ ^]^
iv) In situ characterization methods could be beneficial in investigating the electrocatalytic evolution of polysulfide and the conversion principle in the pSRR and pSOR. Operando in situ observation of MOFs’ growth and their conversion evolution during pyrolysis or posttreatment processes are vital for designing novel polysulfide electrocatalysts. Progressive testing techniques coupling electrochemical tests with in‐situ spectroscopy technologies (such as FT‐IR, Raman spectrum, XPS, etc.) or in situ scanning probe microscopy should be useful in the practical understanding of the kinetic reaction mechanisms in polysulfide catalytic conversion.^[^ ^176^, ^287^ ^]^
v) As the developments of computing sciences, the association of experimental and theoretical researches could lead to new understandings into the unique cooperative effects and induce numerous possibilities for the discoveries and breakthroughs in advanced multimetal‐based MOFs.^[^ ^21^, ^259^, ^288^ ^]^
vi) Systematic researches could be helpful for the construction and preparation of highly effective polysulfide adsorbents and electrocatalysts. Apart from the high adsorption sites, the density and use ratio of catalytic sites, electron transfer, interface performance between various ingredients, active sites, electrolytes, etc., are all significant elements for enhancing the whole electrocatalytic property.^[^ ^230^, ^289^, ^290^, ^291^ ^]^
vii) Highly effective and bifunctional polysulfide catalysts in pSRR and pSOR can be achieved by encapsulating mild active sites into MOFs and MOF‐derived nanostructures.^[^ ^36^, ^37^, ^39^, ^232^ ^]^
viii) To accelerate the redox kinetics of lithium polysulfide and further promote the practical applications of MSBs, more efficient catalytic structures should be adopted, such as MOF‐derived catalysts that integrated with a hierarchical porous structure and catalytic sites with metal compound and single atoms. Besides, the yield and cost of the material should also be considered. Therefore, on the premise of ensuring excellent performance, MOFs with lower price and higher carbon content should be selected, such as ZIF‐8 and ZIF‐67.

Consequently, MOFs will unquestionably persist in inspiring more investigations on polysulfides electrocatalysts for MSBs. From a practical standpoint, MOF‐derived nanostructures with earth‐rich elements can be applied to completely settle the intractable issues suffered by the entire world in energy and the environment. As unremitting research contributions, we will have an excellent opportunity to witness the innovation of advanced technologies in reproducible sources of energy and environmental sciences in the coming future.

## Conflict of Interest

The authors declare no conflict of interest.
